# Prediction and Preparation of Coamorphous Phases of a Bislactam

**DOI:** 10.1021/acs.molpharmaceut.2c00357

**Published:** 2022-06-22

**Authors:** Luke I. Chambers, Osama M. Musa, Jonathan W. Steed

**Affiliations:** † Department of Chemistry, Durham University, Lower Mountjoy, Stockton Road, Durham DH1 3LE, U.K.; ‡ Ashland LLC, 1005 Route 202/206, Bridgewater, New Jersey 08807, United States

**Keywords:** coamorphous, partial least squares-discriminant analysis, predictive modeling, active pharmaceutical ingredients, physical stability, bislactams

## Abstract

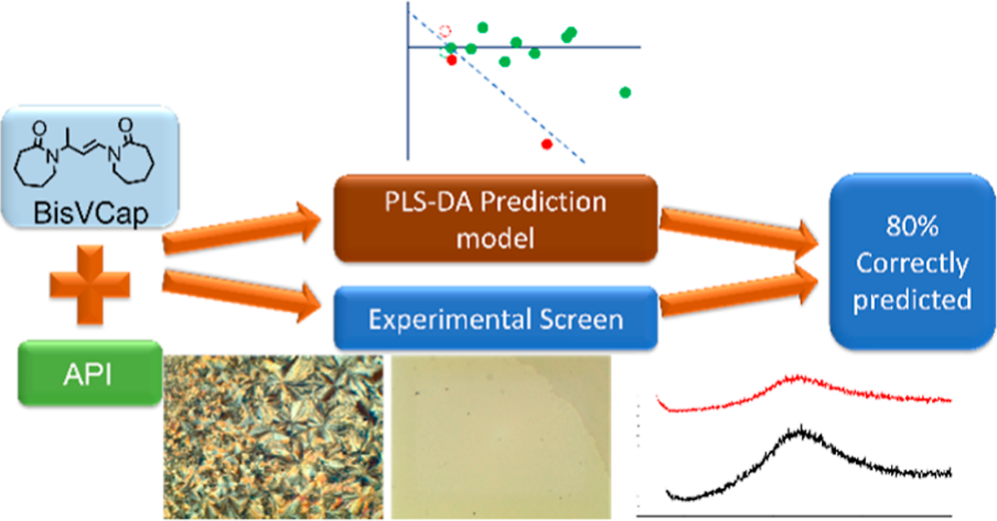

The effectiveness of a partial least squares-discriminant analysis coamorphous prediction model was tested using coamorphous screening data for a promising coamorphous former, the dimer of *N*-vinyl(caprolactam) (bisVCap) with a range of active pharmaceutical ingredients. The prediction model predicted 71% of the systems correctly. An experimental coamorphous screen was performed with this coformer with 13 different active pharmaceutical ingredients, and the results were compared to the predictions from the model. A total of 85% of the systems were correctly predicted. Stability assessments of three coamorphous systems showed that the prediction model score did not strongly correlate with the stability of the coamorphous material. The model performed well with small-molecule coformers, such as bisVCap, despite the difference in structure and properties compared to the amino-acid-based model training set.

## Introduction

The term coamorphous (COAM) system was first introduced by Rades and co-workers in 2009 based on an amorphous material comprising a mixture of ranitidine hydrochloride and indomethacin, which was initially described as a “comilled amorphous sample”.^1^ A COAM material is defined as a mixture of multiple low-molecular-weight components in a single-phase homogeneous amorphous system.^2−6^ The main research interest for COAM systems is for pharmaceutical products; therefore, at least one of the components is usually an active pharmaceutical ingredient (API) and the other is either an inactive coformer or another API.^3,7,8^ COAM materials are of interest to the pharmaceutical industry due to the increased stability of COAM systems compared to the amorphous solids while retaining the dissolution advantage of an amorphous form.^9−11^ The COAM systems also help overcome some of the key issues of polymeric amorphous solid dispersions (PASDs), including the hygroscopicity and large ratio of polymers required to stabilize the API.^12−14^

The stabilization mechanisms of COAM systems include elevated *T*_g_ compared to the pure drug, molecular-level mixing, and intermolecular interactions.^15−18^ COAM systems have been shown to be stable for long periods of time after preparation even at increased temperatures and humidities.^19,20^ COAM systems are known to increase the solubility of a crystalline API in a similar way to cocrystals by the “spring and parachute” effect.^5,21^ However, in some cases, the dissolution rate of the COAM system can be too fast, causing the solution to become supersaturated, which results in the API crystallizing.^22,23^

Previously, we have designed a partial least squares-discriminant analysis (PLS-DA) model using a data set of amino acids with six APIs to predict COAM formation.^24^ The model identifies key parameters, including average molecular weight, the sum of the difference between hydrogen bond donors and acceptors for both components, the excess enthalpy of mixing and of hydrogen bonding, and the difference in the Hansen parameter for hydrogen bonding. In the present work, this model has been applied to a new data set based on a non-amino acid coformer, bisvinylcaprolactam (bisVCap), and applied predictively^24,25^ in the formation of new COAM materials based on this promising coformer, which we have previously shown stabilizes a range of APIs in the COAM state.^26^
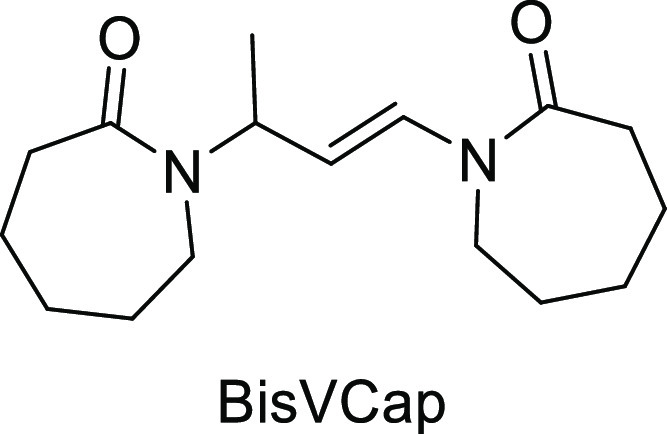


BisVCap is the unsaturated dimer of vinylcaprolactam and contains two highly polar lactam carbonyl groups which act as hydrogen bond acceptor sites.^26−29^ BisVCap is structurally similar to the polymer polyvinylpyrrolidone (PVP), which contains five-membered rings as opposed to bisVCap’s seven-membered rings. One use of PVP is in the formation of PASDs where it stabilizes a range of APIs in an amorphous state.^30−35^ BisVCap is significantly less hygroscopic compared to PVP, with a dynamic vapor sorption (DVS) study showing that amorphous bisVCap displays a mass increase of 1.2% when at 90% relative humidity compared to a 40% mass increase for PVP at 80% relative humidity.^26^

In this work, the reliability of the prediction model based on amino acid coformers was tested with bisVCap by applying the model to our previous COAM screening results and then using the model to predict COAM formation in further experimental systems.^24,26^ Fourier transform infrared spectroscopy (FTIR) was used to analyze the potential interactions between bisVCap and each API to understand the nature of the stabilization. A stability study of three of the resulting COAM systems was performed at two different temperatures to determine if the location of the combination in the PLS-DA prediction space correlates with the stability of the system. The representative COAM system formed from bisVCap furosemide was studied further to test its tolerance to increased drug loading and humidity.

## Experimental Section

### Materials

Piroxicam was purchased from Alfa Aesar (Massachusetts, USA). Aspirin, chloramphenicol, chlorpropamide, indomethacin, ketoprofen, *n*-vinyl caprolactam, paracetamol, phenobarbital, and trifluoroacetic acid were purchased from Merck (Massachusetts, USA). Hexane and acetone were purchased from Thermo Fisher Scientific (Massachusetts, USA). Famotidine was purchased from Tokyo Chemical Industry (Tokyo, Japan). Flurbiprofen, furosemide, mebendazole, and simvastatin were purchased from Fluorochem (Derbyshire, UK). All chemicals were used without further purification.

### Analytical Methods

IR spectra were measured with a PerkinElmer 100 FT-IR spectrometer with an μATR attachment. Data was recorded at a resolution of 4 cm^–1^ for 8 scans over a range of 4000–550 cm^–1^. X-ray powder diffraction (XRPD) measurements were performed using an X’Pert PANalytical PRO X-ray diffractometer (PANalytical, Almelo, The Netherlands) or a Bruker D8 with Cu Kα radiation (1.54187 Å), acceleration voltage and current of 45 kV and 40 mA, respectively. The samples were scanned in the reflectance mode between 2 and 35° 2θ with a scan rate of 0.067335° 2θ/s and a step size of 0.0262606°. The data was collected and analyzed using the software X’Pert Data Collector (PANalytical, Almelo, The Netherlands). ^1^H and {^1^H}^13^C solution NMR spectra were recorded using a Varian Mercury-400 spectrometer, operating at 400 MHz for ^1^H and 100 MHz for ^13^C, chemical shifts are reported in ppm (δ) and referenced to residual protic solvent. Elemental analysis was performed by the University of Durham service using an Exeter CE-440 elemental analyzer. Electrospray mass spectrometry was recorded using a TQD mass spectrometer and an Acquity ultra-performance liquid chromatography. The Acquity photodiode array detector provides absorbance data from 210 to 400 nm. The sample is dissolved in methanol at 1 mg/mL. Hot stage microscopy (HSM) was performed using an Olympus XC50 microscope with a Linkam LTS420 heating stage. Samples were placed onto a glass microscope slide and covered with a thin glass cover slide. Differential scanning calorimetry thermograms were recorded using a PerkinElmer 8500 calorimeter or a PerkinElmer DSC 4000 analyzer, calibrated using an indium standard, with samples accurately weighed (±0.5 mg) into standard aluminum pans. The heating rate was 10 °C min^–1^.

### COSMOquick Calculations

COSMOquick version 1.7 (COSMOlogic GmbH & Co. KG, Leverkusen, Germany) was used to calculate the excess enthalpy of mixing (Δ*H*_mix_) and excess enthalpy of hydrogen bonding (Δ*H*_hb_), of the two-component system. For each component, the following variables were calculated μ, the pseudo-chemical potential of the pure solute and δ*h*, the Hansen parameter for hydrogen bonding. The difference between the API and coformer values were calculated and used as the variables in the PLS-DA.

### Synthesis of BisVCap

*N*-Vinyl caprolactam (30.0 g, 216 mmol) was added to hexane (150 cm^3^) in a two-necked flask with a reflux condenser under nitrogen. The sample was heated to 50 °C to dissolve the *N*-vinyl caprolactam. Trifluoroacetic acid (0.75 cm^3^) was added, and the reaction mixture was heated to 60 °C for 2 h. A white precipitate appeared during the reaction. The solid was removed via vacuum filtration and washed with hexane (3 × 20 mL). The white precipitate was recrystallized twice from acetone to give a white powder. Yield 10.05 g, 36.1 mmol, 33%. Elemental analysis expected for C_16_H_26_N_2_O_2_: C, 69.03; H, 9.41; and N, 10.06. Found: C, 69.10; H, 9.39; and N, 9.99. The analysis is in agreement with published works.^29^^1^H NMR (400 MHz, CDCl_3_): δ 7.26 (dd, *J* = 14.9, 1.7 Hz, 1H, vinyl NCH), 5.43 (qdd, *J* = 6.9, 5.0, 1.7 Hz, 1H, NCH), 5.01 (dd, *J* = 14.9, 5.0 Hz, 1H, vinyl CH), 3.60–3.54 (m, 2H, CH_2_), 3.32–3.12 (m, 2H, CH_2_), 2.68–2.62 (m, 2H, CH_2_), 2.59–2.48 (m, 2H, CH_2_), 1.84–1.47 (m, 12H, CH_2_), 1.27 (d, *J* = 6.9 Hz, 3H, CH_3_). ^13^C NMR (101 MHz, CDCl_3_): δ 175.60, 174.33, 128.13, 110.28, 48.65, 45.32, 43.21, 37.51, 37.17, 30.52, 30.02, 29.54, 29.40, 27.25, 23.41, 16.78. ^13^C{^1^H} SS NMR (101 MHz): δ 174.4, 129.2, 110.1, 50.6, 42.5, 36.4, 31.8, 30.0, 28.3, 25.6, 25.0, 22.3. IR ν = 1667 (C=C), 1641 (C=O), 1622 (C=O) cm^–1^. MS (ESI) *m*/*z*: 278 (M^+^), mp 145 °C.

### HSM Method for COAM Systems

BisVCap and each API were individually heated at 20 °C min^–1^. After the sample was fully melted, it was removed from the HSM and placed on a freezer block to flash cool the sample and prevent crystallization on cooling. The samples were monitored after 24 h using an optical microscope with a polarizer to determine if crystallization had occurred. The same process was repeated using a 1:1 molar ratio of bisVCap and API.

### Comelting for COAM Systems

A 1:1 molar ratio of bisVCap and API was heated in a vial to a few degrees above the highest melting point out of the API or bisVCap (mp 145 °C). The mixture was held at this temperature for 10 min and then, it was rapidly cooled by submerging the vial into dry ice and acetone. The mixtures were then analyzed via XRPD and FTIR to check if the sample was amorphous.

### Rapid Solvent Evaporation for COAM Systems

The chosen ratio of bisVCap and API was dissolved in the minimum amount of acetone. The solvent was rapidly removed under reduced pressure on a water bath at 60 °C. The mixtures were then analyzed via XRPD and FTIR to check if the sample was amorphous.

### Stability Test at Different Temperatures

COAM samples of bisVCap with indomethacin, paracetamol, and simvastatin in a 1:1 molar ratio were produced by rapid solvent evaporation (RSE). The samples were either stored at ∼20 or 3 °C. The samples were characterized by XRPD and FTIR after 1 and 2 weeks.

### Varying Humidity Stability Test

A COAM sample of bisVCap and furosemide in a 1:1 molar ratio was produced via RSE. Five 50 mg samples were placed in a vial and stored in five different humidity environments including 0, 11, 33, 75, and 100% RH using relevant saturated salt solutions. The samples were analyzed by XRPD and FTIR after 7 and 28 days.

## Results and Discussion

### Testing the Model against Previous COAM Screening Data

The results of the COAM screen of bisVCap with a range of APIs previously published by Goodwin et al.^26^ were analyzed using the amino acid-based PLS-DA COAM formation prediction model to investigate the effectiveness of the model with bisVCap coformers.^24^ The COAM data involved 12 bisVCap API systems produced by comelting (CM) the two components and then cooling to room temperature. Three of the APIs used (ibuprofen, tolfenamic acid, and ethionamide) decompose on melting; therefore, these systems were removed from this study. Mexiletine and metformin were also removed from the study because they are hydrochloride salts, and the prediction model has not been designed to consider ionic compounds. From the remaining seven systems, the experimental results showed that three form COAM systems while the other four do not (Table 1).

**Table 1:** Results of an Experimental COAM Screen from Goodwin et al.^26^ Compared to the Predicted Result Using the Prediction Model from Chambers et al.^24^

API	label	experimental COAM	predicted COAM	correctly predicted
benzocaine	BENZ	N	N	Y
caffeine	CAFF	N	N	Y
carbamazepine	CARB	Y	N	N
carisoprodol	CARI	Y	N	N
dopamine	DOPA	N	N	Y
isoniazid	ISON	N	N	Y
valsartan	VALS	Y	Y	Y

**Figure 1:**
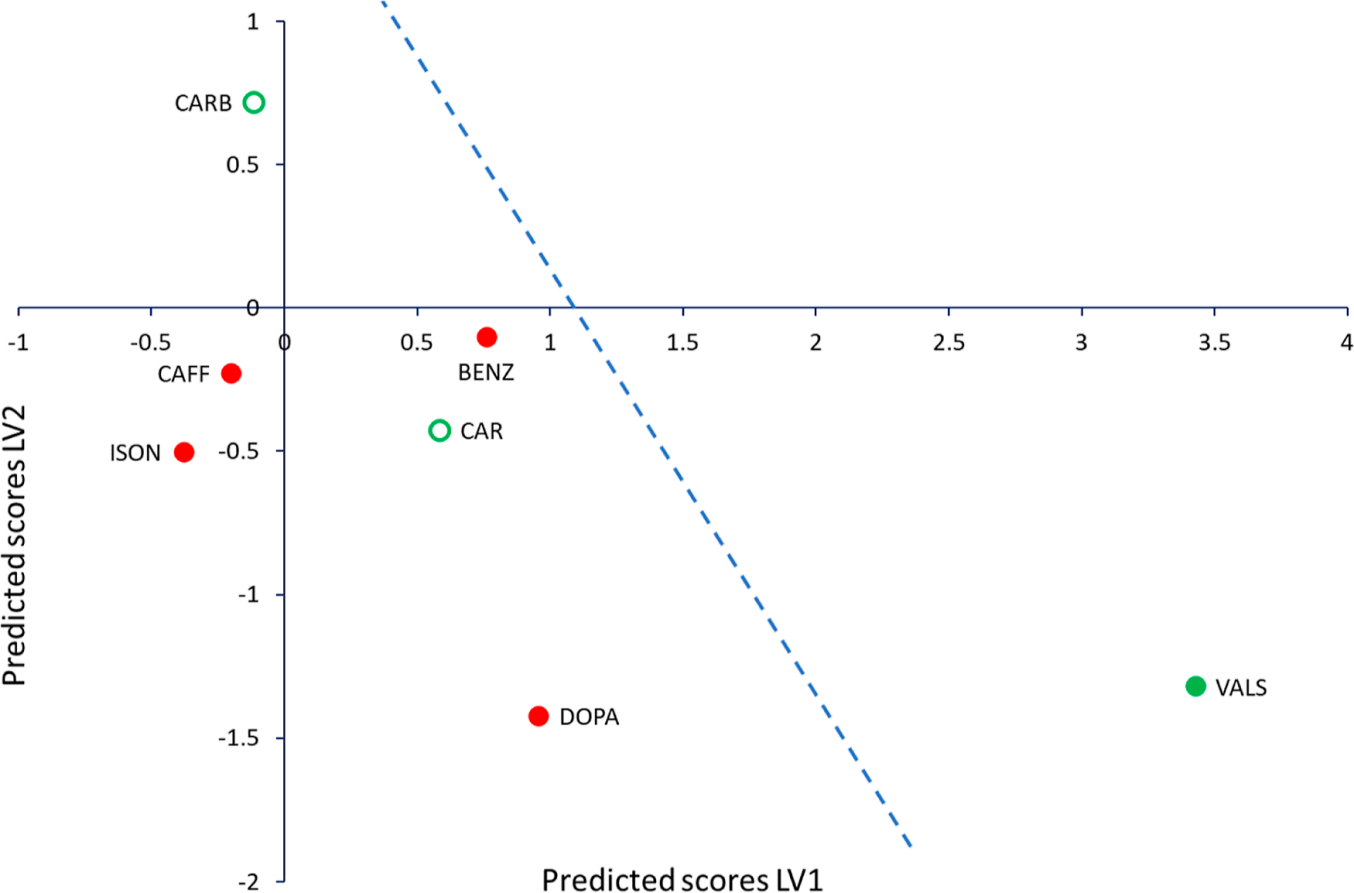
PLS-DA score scatter plot of latent variables (LV) 1 and 2. The color of the markers displays the results of the previous experimental screen^26^ with red markers indicating not COAM systems and green markers indicating COAM systems. Samples incorrectly predicted by the prediction model are shown as hollow circles.^24^ The blue dashed line shows the predicted separation line between COAM and not COAM systems based on the PLS-DA calculation for visualization purposes.

The score plot visually represents how close each system is to the predicted crossover line between COAM and non-COAM systems (Figure 1). The model correctly predicts 71% of this sample set with a Fisher’s probability of 0.43.^36^ The two systems incorrectly predicted are carbamazepine and carisoprodol, which were experimentally COAM but are predicted not to be COAM. The FTIR spectrum of the COAM systems of bisVCap with carbamazepine and carisoprodol suggests reduced hydrogen bonding strength to the API carbonyl group indicating the formation of an amorphous state because the carbonyl peaks shift to a higher wavenumber compared to the crystalline API. However, the bisVCap carbonyl peaks broaden but do not shift to a lower wavenumber, indicating the bisVCap carbonyl groups are not forming new hydrogen bonds with the API.^26^ The incorrect prediction of carbamazepine and carisoprodol could therefore indicate the model is based around the potential formation of stabilizing intermolecular bonds between the two components, which are not present in this case.

### COAM Systems Based on Unknown BisVCap API Combinations

To determine the broader applicability of the PLS-DA model a range of 13 additional APIs were selected based on a range of chemical functionality and paired with bisVCap. The PLS-DA prediction model predicts that 10 out of these 13 combinations should form a coamorphous phase (Table 2). To test these predictions, bisVCap was mixed in a 1:1 molar ratio with each API and melt quenched using HSM. The mixtures were monitored for signs of recrystallization after 24 h using an optical microscope after storage at ambient conditions (Table S1). The HSM screen showed that bisVCap by itself recrystallizes on cooling; however, when mixed with any of the APIs no recrystallization occurs, showing these 13 APIs prevent bisVCap crystallization. Pure famotidine, furosemide, mebendazole, and piroxicam all undergo decomposition when heated to near their melting point. Similar decomposition occurs for the 1:1 mixtures of bisVCap with famotidine, mebendazole, and piroxicam; however, the furosemide mixture with bisVCap appears to remain amorphous and minimal decomposition occurs on melting. Pure aspirin, chlorpropamide, flurbiprofen, paracetamol, and phenobarbital begin to recrystallize after 24 h; however, the mixtures with bisVCap remain amorphous, suggesting bisVCap can stabilize the systems in an amorphous state. Pure chloramphenicol, indomethacin, ketoprofen, and simvastatin remain amorphous after 24 h, and mixtures of these APIs with bisVCap are also amorphous suggesting the interactions between API and bisVCap are preventing the bisVCap from recrystallizing.

**Table 2:** Predictions and Experimental Results of the COAM Screen of 13 APIs with bisVCap^a^

API	label	prediction (COAM value)	HSM	DSC (*T*_g_/°C)	CM XRPD	RSE XRPD	correctly predicted
aspirin	ASPR	Y (0.864)	Y	Y (−19)	Y	Y	Y
chloramphenicol	CHPL	Y (0.682)	Y	Y (31)	Y	Y	Y
chlorpropamide	CHPD	Y (0.500)	Y	Y (−4)	Y	Y	Y
famotidine	FAMO	N (0.463)	D	D	D	N	Y
flurbiprofen	FLURB	Y (1.096)	Y	Y (−5)	Y	Y	Y
furosemide	FUR	Y (1.091)	Y	Y (35)	Y	Y	Y
indomethacin	INDO	Y (1.142)	Y	Y (16)	Y	Y	Y
ketoprofen	KETO	Y (0.833)	Y	Y (−5)	Y	Y	Y
mebendazole	MEB	Y (0.556)	D	D	D	N	N
paracetamol	PARA	N (0.450)	Y	Y (19)	Y	Y	N
phenobarbital	PHB	Y (0.588)	Y	Y (28)	Y	Y	Y
piroxicam	PIRO	N (0.443)	D	D	D	N	Y
simvastatin	SIM	Y (0.751)	Y	Y (12.5)	Y	Y	Y

a The prediction includes the predicted COAM value. The experimental COAM screen includes the results from initial HSM screening, the *T*_g_ from DSC, and the XRPD analysis of both CM and rapid sovlent evaporation (RSE) samples. Y indicates the samples were COAM, N indicates the samples were not COAM, and D indicates the samples decomposed. The predicted result was also compared with the experimental results to determine if the prediction was correct.

The combination of bisVCap and the 13 APIs was investigated using both CM and rapid solvent evaporation (RSE) to allow further analysis by XRPD and FTIR (Figure S3, Table 2). The two methods CM and RSE were selected to help cover a larger experimental space and to remove any issues caused by the decomposition of samples during CM or difficulty dissolving the samples in some solvents for RSE. Ball milling was not used because bisVCap has a low glass transition temperature (*T*_g_) and milling above the *T*_g_ usually leads to polymorphic transformation instead of amorphization.^37,38^ The XRPD patterns (Figure S2, Table 2) for the RSE samples show the same results as observed in the HSM screen with the 10 systems that did not decompose producing COAM systems using both CM and RSE. The three systems that decomposed via HSM (famotidine, mebendazole, and piroxicam) did not produce a COAM system by RSE.

**Figure 2:**
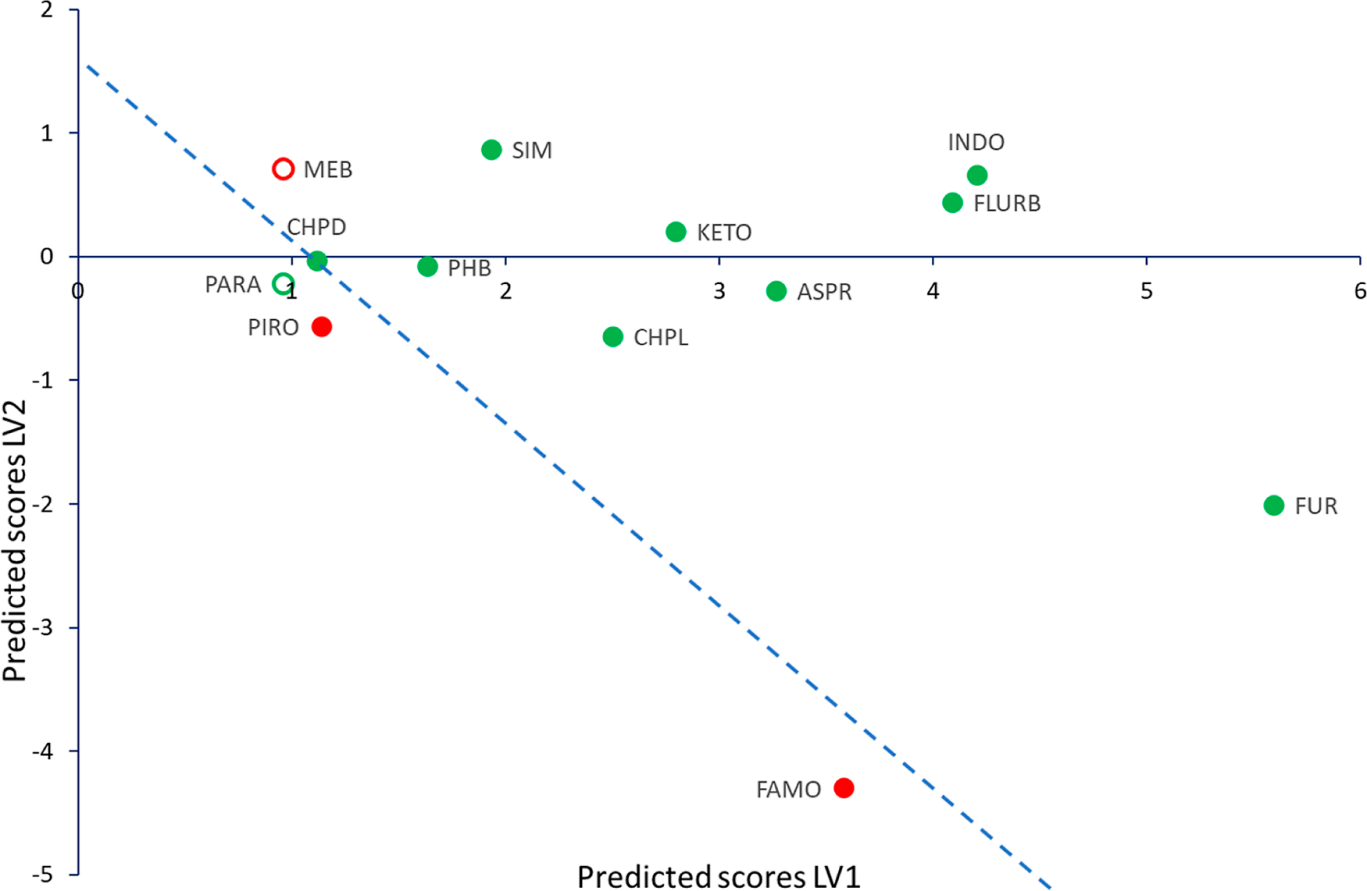
PLS-DA score scatter plot of LV 1 and 2. The color of the markers displays the results of the experimental screen with red markers indicating not COAM systems and green markers indicating COAM systems. Samples incorrectly predicted by the prediction model are shown as hollow circles.^24^ The blue dashed line shows the predicted separation line systems based on the PLS-DA calculation for visualization purposes.

The 10 systems found to be amorphous by HSM and XRPD were analyzed as physical mixtures, produced by gently grinding the two components together with a spatula, by DSC to determine the *T*_g_ and check for decomposition (Figure S1, Table 2). All of the DSC traces display the API and bisVCap melting in the first heating cycle, no recrystallization during a cooling cycle and a clear glass transition in the second heating cycle. Each of the 10 systems only display a single *T*_g_ confirming the samples as COAM and not a mix of two amorphous materials. Comparing the experimental results with the predictions of the model shows that 11 of the 13 samples were correctly predicted (Table 2). The overall prediction success is therefore 85% suggesting the model is good at predicting bisVCap COAM systems, which is surprising given the chemically different natures of the amino acid coformers used in the training data set compared to bisVCap. While amino acids have good hydrogen bond donor and acceptor groups, bisVCap has only very weak hydrogen bond donor capability.^29^ The PLS-DA score scatter plot shows that the two incorrectly predicted systems paracetamol and mebendazole are close to the prediction separation line (Figure 2). Mebendazole in particular decomposes during CM and is poorly soluble in acetone during RSE and hence, alternative COAM preparation methods may prove successful.

### Stabilizing Interactions in COAM Systems

FTIR spectroscopy was used to understand the interactions in the COAM systems (Figure S3). The carbonyl stretching bands for solid bisVCap occur at 1622 and 1640 cm^–1^ and its X-ray crystal structure demonstrates that they do not form any strong hydrogen bonding interactions.^29^ In all the non-COAM systems (Table 2), the position of the bisVCap carbonyl bands are unchanged suggesting no new interactions are occurring; however, in all the COAM systems, the bisVCap carbonyl peaks shift to a lower wavenumber and broaden implying that the bisVCap carbonyl groups are forming stronger hydrogen bonds causing the weakening of the C=O bond.^15,39,40^ The FTIR spectra of the COAM systems show clear changes in the APIs bands as well indicating the disruption of the hydrogen bonding structure of the parent single component phase. The FTIR data suggest bisVCap stabilizes the COAM system by acting as a hydrogen bond acceptor with the APIs containing hydrogen bond donor groups.

### Stability Study at Different Temperatures

**Figure 3:**
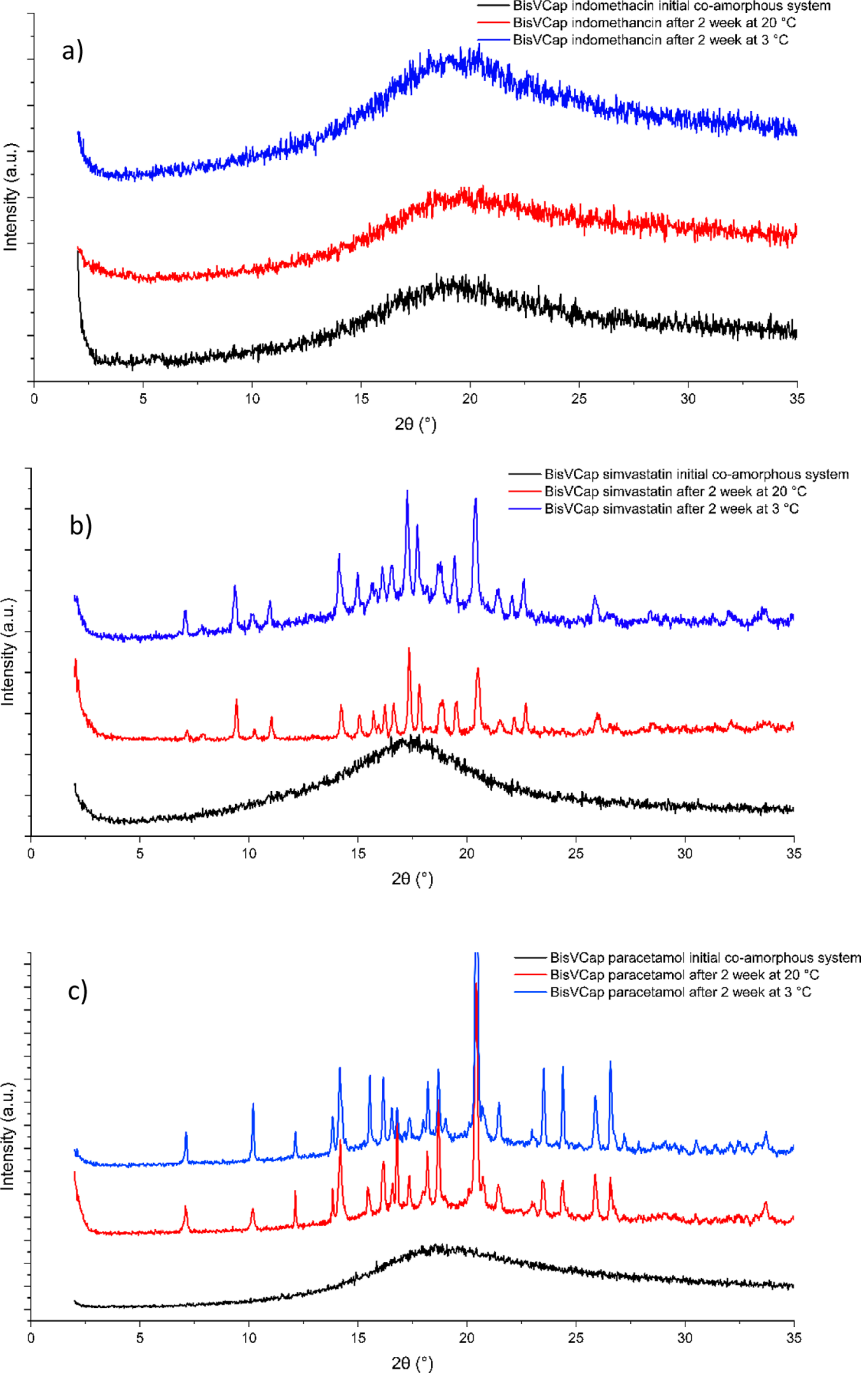
XRPD traces of COAM samples of bisVCap with (a) indomethacin, (b) simvastatin, and (c) paracetamol. The initial COAM samples made by RSE are shown in black. The XRPD traces are shown after 2 weeks when stored at both 20 °C (red) and 3 °C (blue).

The stability of three of the experimentally generated COAM systems (Table 2) was tested to determine if the predicted COAM PLS-DA score correlates with the stability of the COAM product. The three systems selected were bisVCap with paracetamol, simvastatin, and indomethacin, with corresponding COAM values of 0.450, 0.751, and 1.142. Therefore, if the COAM score is an indication of stability, it would be expected that the COAM material formed from bisVCap and indomethacin would be the most stable and the one with paracetamol the least stable. These three COAM materials were produced by RSE and stored in sealed vials at both 20 and 3 °C and analyzed after 2 weeks by XRPD and FTIR spectroscopy. The XRPD (Figure 3) and FTIR (Figure S4) data shows that the indomethacin material remains COAM after 2 weeks; however, both simvastatin and paracetamol have recrystallized. The XRPD pattern of the recrystallized COAM simvastatin bisVCap system after 2 weeks at both temperatures (Figure 3b) showed the crystallization of bisVCap and the formation of the form I polymorph of simvastatin, which is the thermodynamically stable polymorph above 0 °C.^41,42^ A similar process was observed for the paracetamol bisVCap system (Figure 3c) with the system separately recrystallizing into bisVCap and the form I polymorph of paracetamol, which is the thermodynamically stable form at the tested conditions.^43,44^ The XRPD patterns for the samples stored at 20 °C showed complete conversion, while some amorphous content remained in each of the samples stored at 3 °C. The formation of the thermodynamically stable polymorph of the simvastatin and paracetamol suggests bisVCap only slows the crystallization rate but does not affect the crystallization process. The apparent greater stability of the indomethacin COAM phase correlates with the higher COAM score from the PLS-DA model.

**Figure 4:**
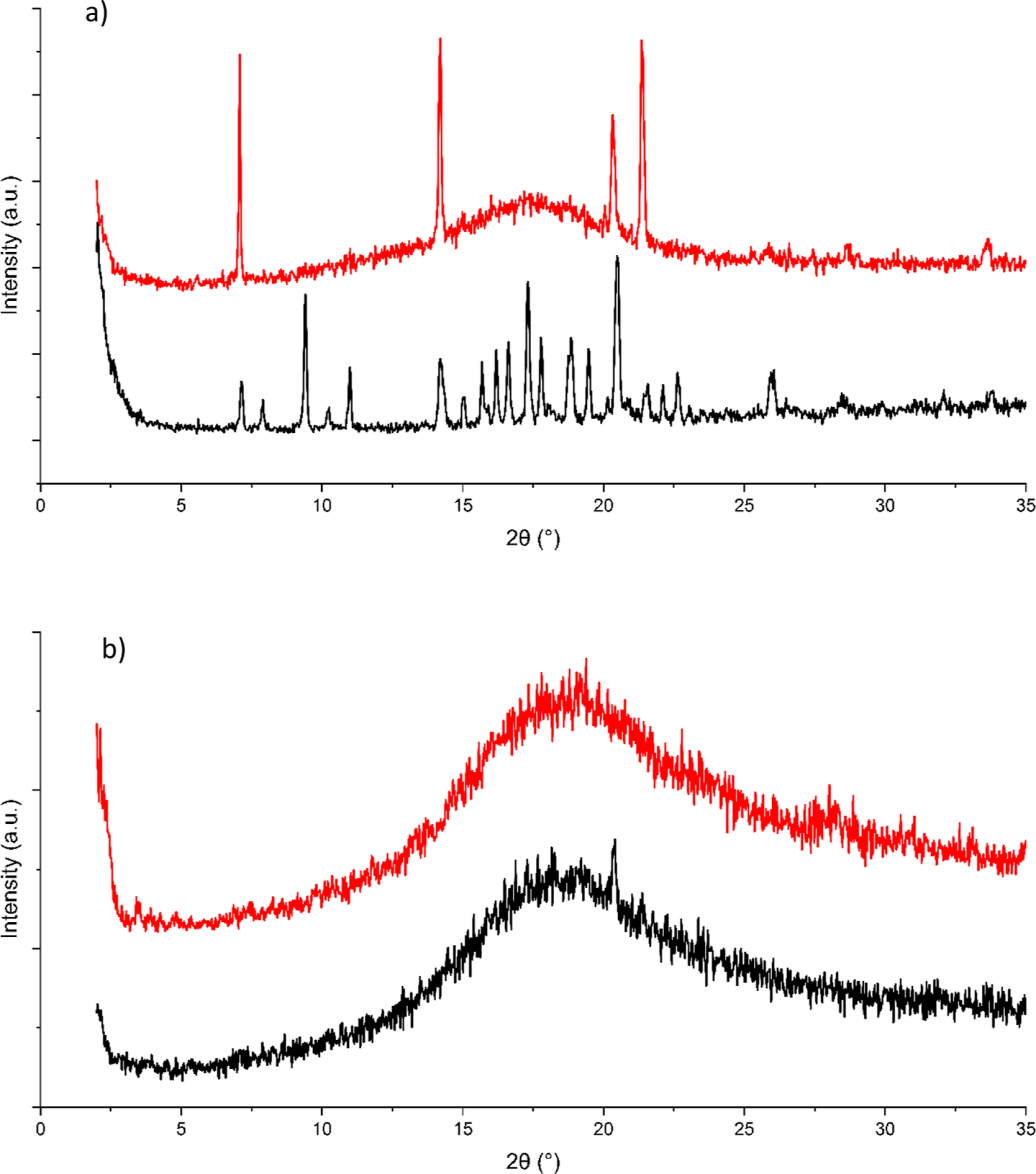
XRPD traces of COAM samples of bisVCap with (a) simvastatin and (b) paracetamol. The XRPD traces are shown after 1 week when stored at ∼20 °C (black) and 3 °C (red).

A second stability experiment with a shorter timescale was carried out with bisVCap simvastatin and paracetamol COAM phases. The two systems were produced by RSE and samples were stored for 1 week at both 20 and 3 °C. The resulting samples were analyzed by XRPD (Figure 4) and FTIR (Figure S5). The XRPD pattern shows the simvastatin COAM material stored at 20 °C crystallizes into bisVCap and the form I polymorph of simvastatin; however, the sample stored at 3 °C proved to be a mixture of crystalline bisVCap and amorphous material indicating that it is the coformer that crystallizes first rather than both components simultaneously. In the system with paracetamol, no recrystallization had occurred after 1 week at 3 °C and only slight recrystallization had occurred for the sample stored at 20 °C. The paracetamol COAM system is, therefore, assumed to be more kinetically stable than the simvastatin COAM system, which is the opposite of the prediction based on the COAM values, suggesting that the PLS-DA score has limited use in predicting stability order.

### Increasing the Ratio of Furosemide to BisVCap

**Figure 5:**
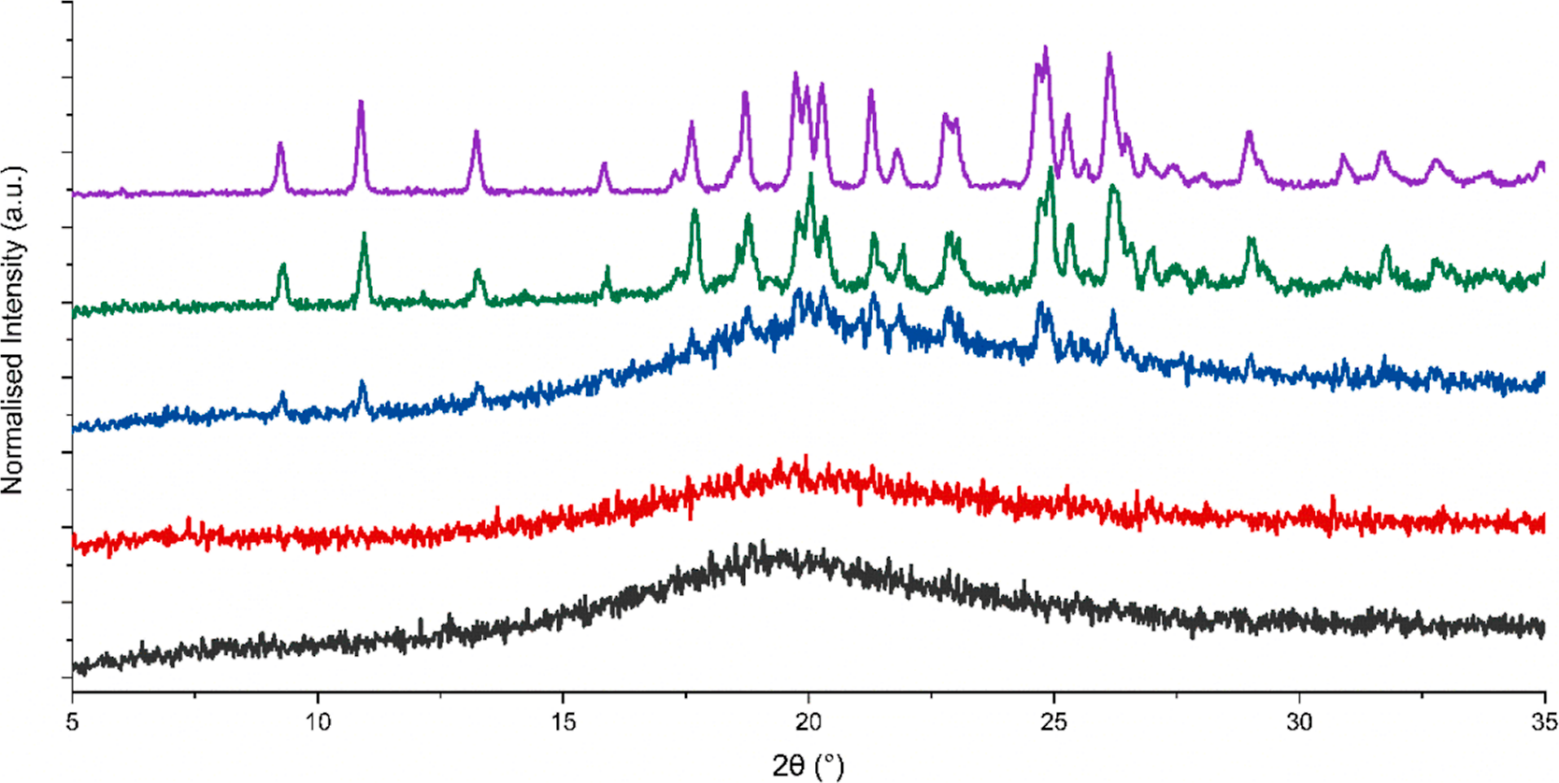
XRPD diffractograms of bisVCap with furosemide after RSE in a 1:1 (black), 1:2 (red), 1:3 (blue), and 1:4 ratio (green). Pure form II furosemide after RSE is shown in purple.

**Figure 6:**
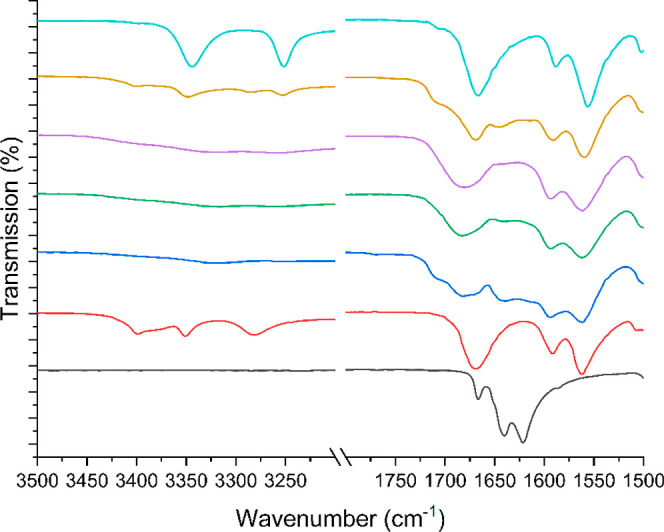
FTIR spectra for the increased ratio study of bisVCap and furosemide showing the carbonyl and alcohol region. The spectra display pure bisVCap (black) and pure furosemide (red). The bisVCap furosemide systems were made by RSE at different ratios with 1:1 in blue, 1:2 in green, 1:3 in purple, and 1:4 in light brown. The furosemide systems which underwent RSE is also shown in cyan.

The COAM system formed from furosemide and bisVCap has the highest *T*_g_ (Table 2) and occurs as a free-flowing light-yellow powder, whereas the other systems are in the form of viscous pastes. The COAM system produced in the screening experiments has a 1:1 molar ratio of bisVCap to furosemide. However, COAM systems have been shown to form with increased ratios of API to a coformer and it is of interest to know how much API loading the COAM phase can tolerate.^45^ Therefore, the preparation of the COAM phase using RSE was repeated using 1:2, 1:3, and 1:4 ratios of bisVCap to furosemide. The RSE process was also repeated with a sample of furosemide in the absence of coformer to determine if the bisVCap is necessary to make the system amorphous. The systems were analyzed by XRPD and FTIR to determine the crystallinity and solid form of the resulting products due to the speed of the results obtained allowing the results to capture any unstable coamorphous states. The XRPD data (Figure 5) shows the bisVCap furosemide systems prepared by RSE remain amorphous up to a 1:2 ratio. The higher ratio samples undergo recrystallization into the form II polymorph of furosemide, which is the polymorph usually formed from evaporation under reduced pressure of a furosemide acetone solution.^46^ The FTIR spectra (Figure 6) of all of the samples show that the bisVCap does not recrystallize as the carbonyl peaks do not return to the original position. The peaks in the XRPD diffractogram of the 1:4 ratio sample can all be assigned to form II furosemide. Therefore, the 1:2 ratio seems to be the API loading limit before crystallization begins. The 1:3 material displayed a significant amorphous halo suggesting a mixture of COAM material and excess crystalline furosemide.

### Stability Study on BisVCap and Furosemide at Different Humidities

**Figure 7:**
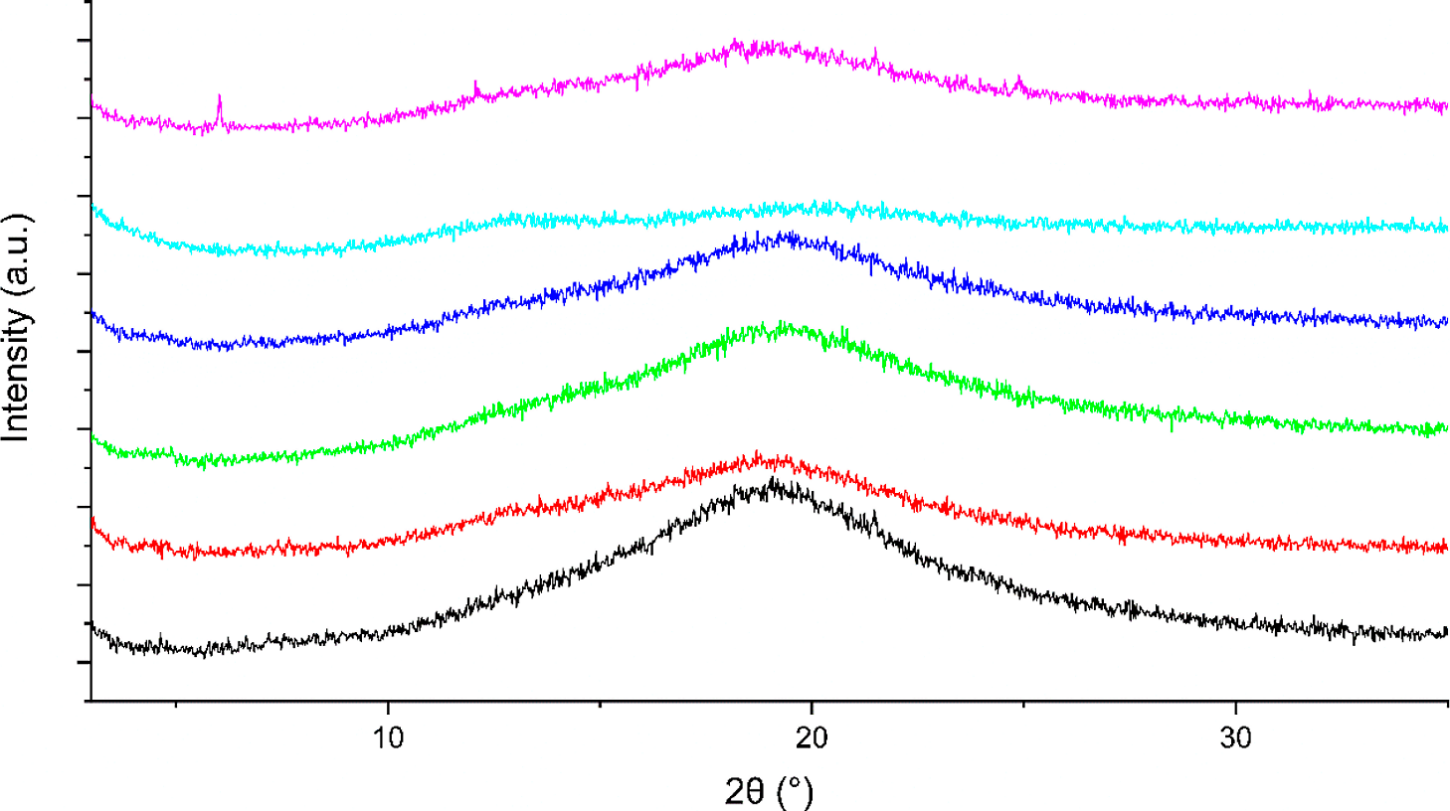
XRPD traces of a COAM bisVCap furosemide material made by RSE. The initial system is shown in black. The system was stored for 7 day at 0% RH (red), 11% RH (green), 33% RH (blue), 75% RH (cyan), and 100% RH (pink).

To understand the effect of humidity on the bisVCap COAM systems a 1:1 mixture of bisVCap and furosemide was prepared by RSE and stored in five different humidity environments (0, 11, 33, 75, and 100%). The systems were analyzed by XRPD and FTIR after seven days and 28 days to see the effect of the different humidity environments.^47^ The initial XRPD data (Figure 7) shows the system is amorphous. After 7 days the material stored at 0, 11, and 33% RH remained unchanged by XRPD (Figure 7) and FTIR (Figure S6), and the material retained the appearance of a free flowing powder. The material stored at 75% RH appears to remain amorphous by both FTIR (Figure S6) and XRPD (Figure 7); however, the material changed from a powder to a glass-like film. No recrystallization was observed by polarized optical microscopy indicating the system remain amorphous even after absorption of water. The material stored at 100% RH turned into a thick paste and started to recrystallize as shown by XRPD with small Bragg peaks observed corresponding to the form I polymorph of furosemide which is the thermodynamically stable form under ambient conditions however, the FTIR spectrum remained unchanged suggesting only a small amount of the sample has recrystallized.^48^

**Figure 8:**
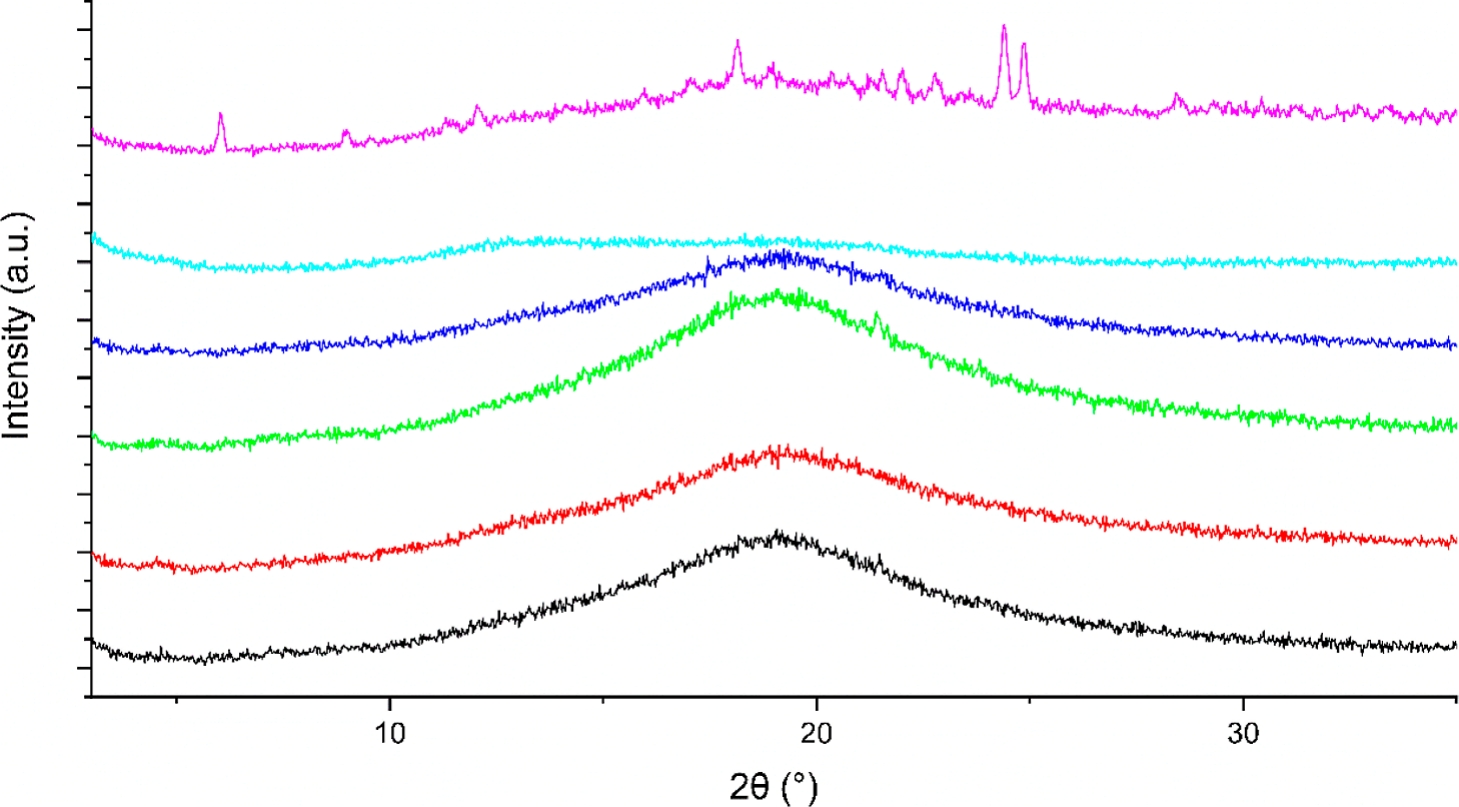
XRPD traces of a COAM bisVCap furosemide system made via RSE. The initial system is shown in black. The system was stored for 28 days at 0% RH (red), 11% RH (green), 33% RH (blue), 75% RH (cyan), and 100% RH (pink).

After storage for 28 days in the five different humidity environments, the materials stored at 0 to 75% RH remained unchanged with no indications of recrystallization by FTIR (Figure S7) or XRPD (Figure 8). The material stored at 100% RH underwent significant crystallization as observed by FTIR with the appearance of sharper peaks in the 3200–3500 cm^–1^ region corresponding to the N–H stretching bands and XRPD displaying Bragg peaks matching the form I polymorph as well as some remaining amorphous halo.^48^ Overall, it appears the 1:1 bisVCap furosemide system remains stable up to 75% RH.

## Conclusions

In conclusion, the PLS-DA prediction model was tested against the results of a previously published COAM screening of bisVCap with a range of APIs, successfully predicting five out of seven systems. The model was then used to predict the outcomes of COAM screening of bisVCap with 13 further APIs expected to form both COAM and non-COAM systems. Each of the mixtures was experimentally prepared by both CM and rapid solvent evaporation. The PLS-DA model successfully predicted the outcome in 11 of the 13 systems. The FTIR data indicates that bisVCap stabilizes the COAM systems by the formation of hydrogen bonds with the two carbonyl groups disrupting the intermolecular hydrogen bonding in the pure API.

The stability of three COAM systems of bisVCap with indomethacin, paracetamol, and simvastatin was analyzed at two different storage temperatures to determine if the prediction model COAM score correlates with the stability of the COAM phases. The stability assessment showed that indomethacin was the most stable agreeing with the model; however, the paracetamol system proved more stable than simvastatin suggesting the prediction model score is not strongly correlated with stability.

The stability of the COAM system as a function of API loading showed that up to a 2:1 ratio of API to bisVCap remains amorphous, with crystallization of the API thereafter. This may be because of two hydrogen bond acceptor carbonyl groups on the coformer. A variable humidity study showed that the 1:1 bisVCap furosemide system remains stable for at least 28 days when stored at 75% RH. The bisVCap coformer is a remarkably effective way of stabilizing coamorphous phases with a range of APIs and in 85% of cases the formation of a COAM phase is successfully predicted by the PLS-DA model despite the very different systems used to train it.

## Supporting Information Available

IR spectra, XRPD patterns, photographs of samples, DSC traces, and underlying data for IR, XRPD, and DSC experiments (PDF)
