# Supplementary material for: Prediction and Preparation of Coamorphous Phases of a Bislactam
Source: Mol Pharm. 2022 Jun 22;19(7):2651–61. doi: 10.1021/acs.molpharmaceut.2c00357 (PMC9257746; doi:10.1021/acs.molpharmaceut.2c00357)
Supplement: Supplementary file 1 — mp2c00357_si_001.pdf [file mp2c00357_si_001.pdf]

Prediction and Preparation of Co-amorphous Phases of a Bislactam

Luke I. Chambers, Osama M. Musa, Jonathan W. Steed*.

Supplementary material

Table S1: Optical micrographs of samples melted using an HSM after 24 hours. The first column shows the pure APIs, and the second column shows the APIs in a 1:1 molar ratio with bisVCap.

Figure S1: The heat/cool/heat DSC thermograms of 1:1 mixtures of the APIs with bisVCap with exotherms up. The different APIs are a) aspirin, b) chloramphenicol, c) chlorpropamide, d) flurbiprofen, e) furosemide, f) indomethacin, g) ketoprofen, h) paracetamol, i) phenobarbital and j) simvastatin. The black trace displays the initial heat cycle, followed by the cooling cycle in red and the second heat cycle in green. The T_g is highlighted with a blue box on each thermogram.

Figure S2: The XRPD diffractograms for the COAM screen of bisVCap with 13 APIs. The CM samples are shown in black and the RSE samples are in red. The different APIs are a) aspirin, b) chloramphenicol, c) chlorpropamide, d) famotidine, e) flurbiprofen, f) furosemide, g) indomethacin, h) ketoprofen, i) mebendazole, j) paracetamol, k) phenobarbital, l) piroxicam, and m) simvastatin. Famotidine, mebendazole and piroxicam only have an RSE trace due to the system decomposing when undergoing CM.

Figure S3: The FTIR spectra for the COAM screen of bisVCap with 13 APIs. BisVCap is shown in black, the pure API in red, the CM sample in blue and the RSE sample in green. The different APIs are a) aspirin, b) chloramphenicol, c) chlorpropamide, d) famotidine, e) flurbiprofen, f) furosemide, g) indomethacin, h) ketoprofen, i) mebendazole, j) paracetamol, k) phenobarbital, l) piroxicam, and m) simvastatin. The famotidine, mebendazole and piroxicam spectra only display an RSE sample due to the system decomposing when undergoing CM.

Figure S4: The FTIR spectra of COAM samples of bisVCap with a) indomethacin, b) simvastatin and c) paracetamol. The initial COAM sample made by RSE is shown in black. The FTIR spectra are shown after two weeks when stored at ~20 °C (red) and 3 °C (green).

Figure S5: The FTIR spectra of COAM samples of bisVCap with a) simvastatin and b) paracetamol. The initial FTIR spectra after RSE is shown in black. The FTIR spectra are shown after one week when stored at ~20 °C (red) and 3 °C (green).

Figure S6: The FTIR spectra of a COAM bisVCap furosemide system made by RSE. Pure bisVCap is shown in black, pure furosemide in red and the initial bisVCap furosemide COAM system in green. The system was stored for seven days at 0% RH (blue), 11% RH (cyan), 33% RH (pink), 75% RH (yellow) and 100% RH (brown).

Figure S7: The FTIR spectra of a COAM bisVCap furosemide system made via RSE. Pure bisVCap is shown in black, pure furosemide in red and the initial bisVCap furosemide COAM system in green. The system was stored for 28 days at 0% RH (blue), 11% RH (cyan), 33% RH (pink), 75% RH (yellow) and 100% RH (brown).

Table S1: Optical micrographs of samples melted using an HSM after 24 hours. The first column shows the pure APIs, and the second column shows the APIs in a 1:1 molar ratio with bisVCap.

API	Pure API	API with bisVCap
BisVCap	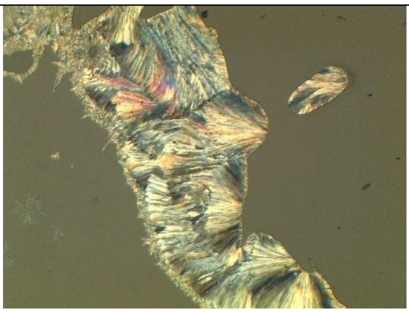	n/a
Aspirin	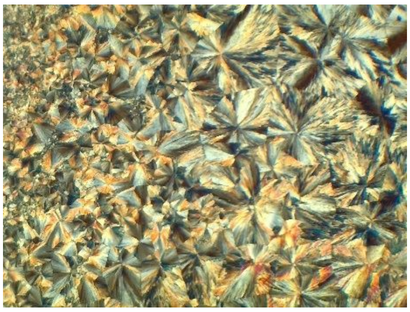	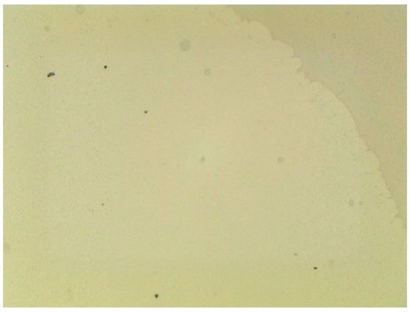
Chloramphenicol	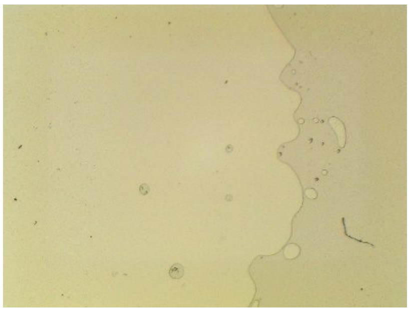	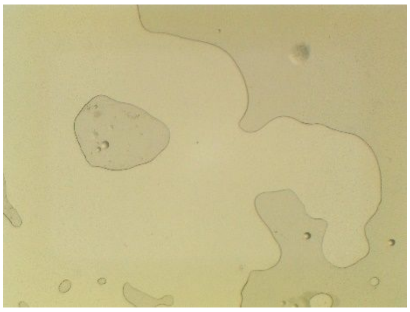
Chlorpropamide	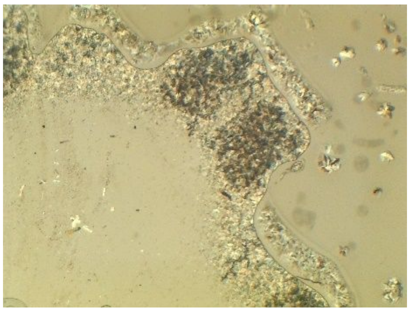	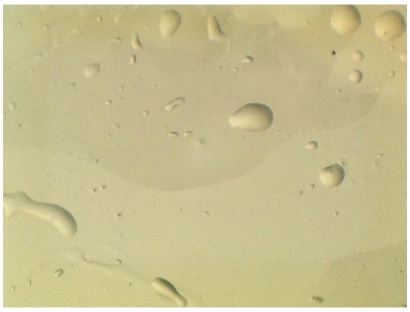

API	Pure API	API with bisVCap
Famotidine	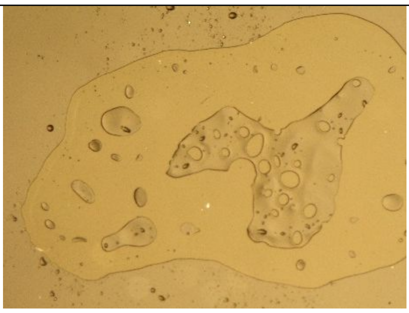	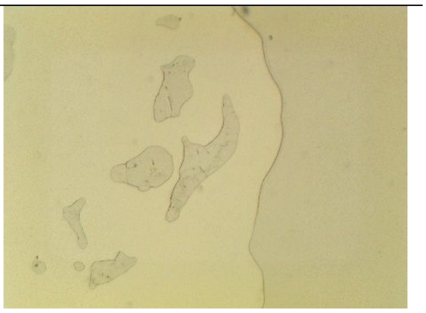
Flurbiprofen	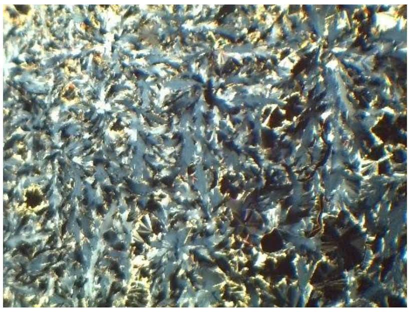	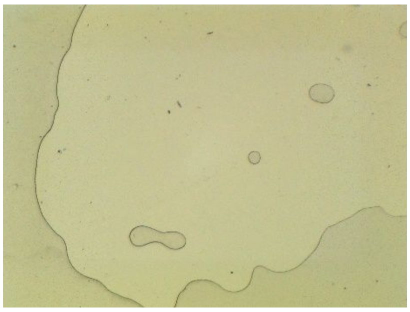
Furosemide	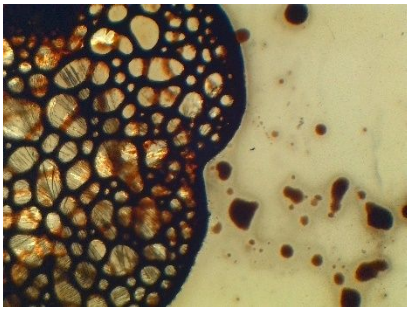	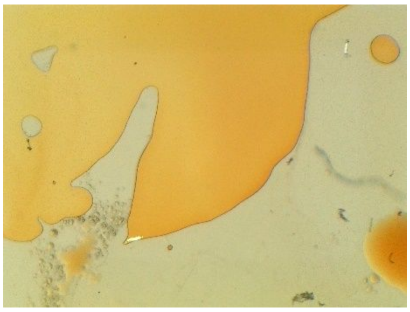
Indomethacin	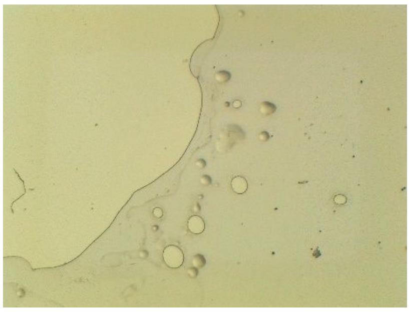	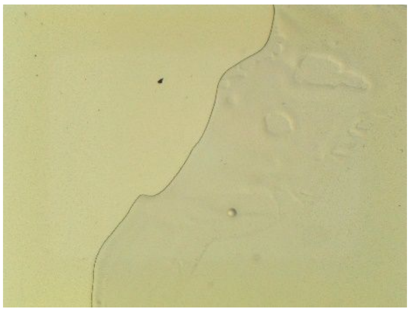
Ketoprofen	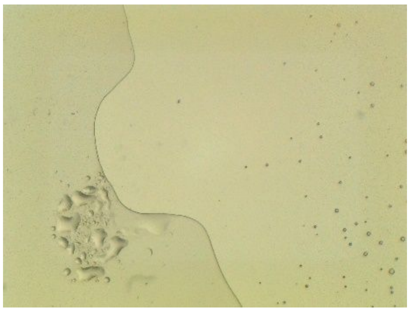	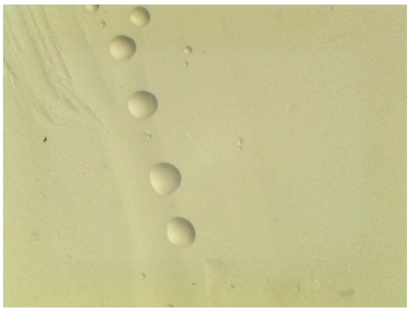

API	Pure API	API with bisVCap
Mebendazole	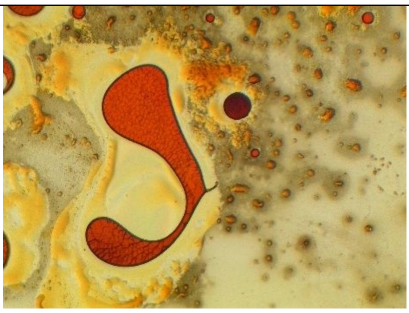	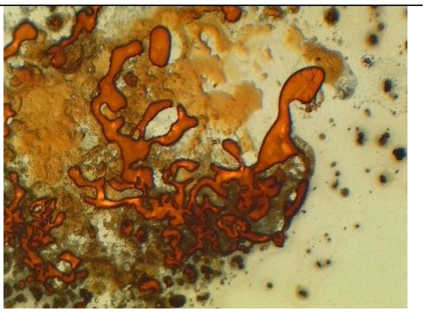
Paracetamol	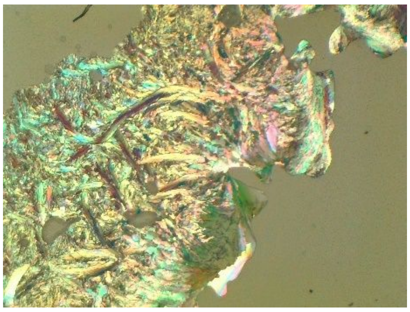	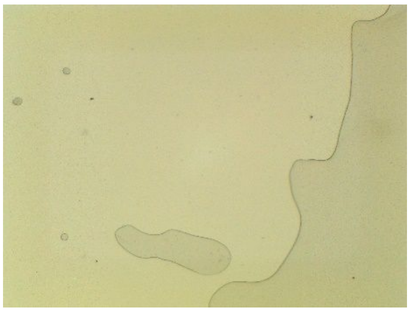
Phenobarbital	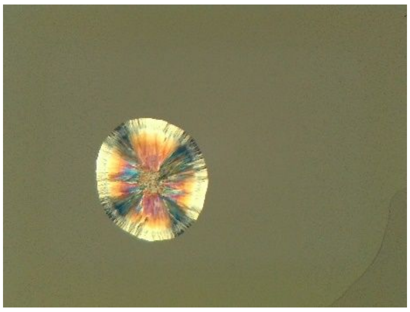	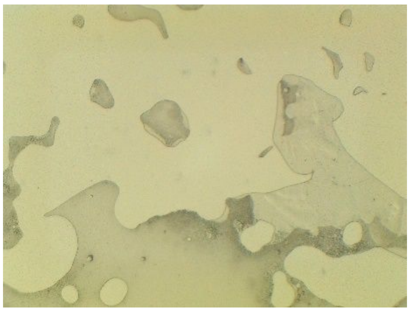
Piroxicam	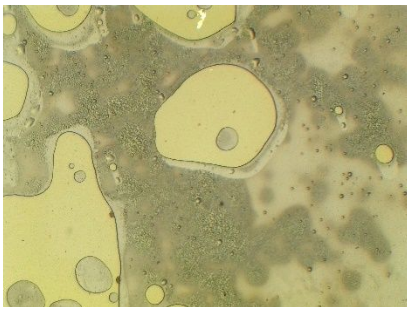	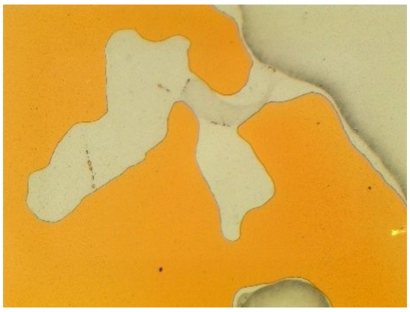
Simvastatin	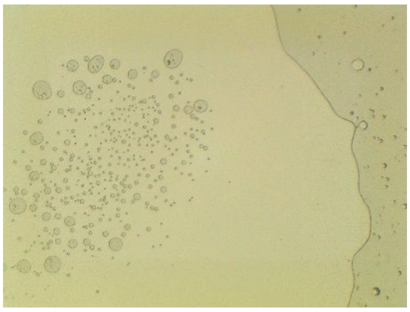	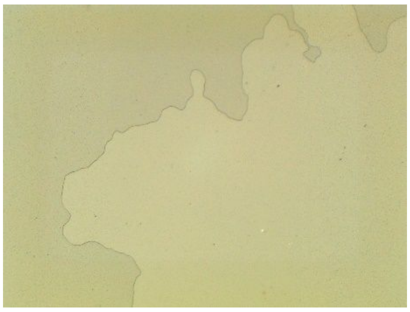

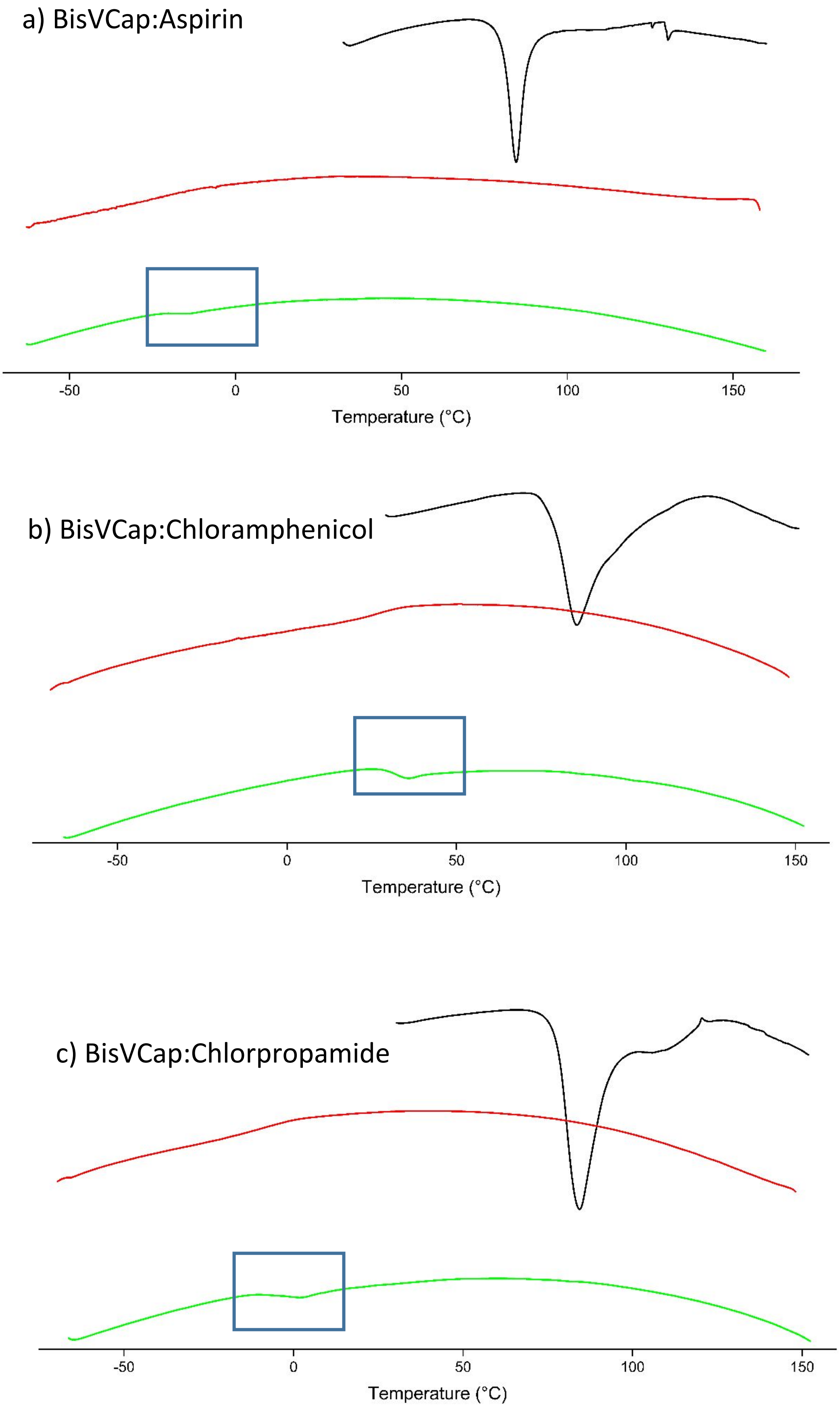
a) BisVCap:Aspirin
-50
0
50
100
150
Temperature (°C)
b) BisVCap:Chloramphenicol
-50
0
50
100
150
Temperature (°C)
c) BisVCap:Chlorpropamide
-50
0
50
100
150
Temperature (°C)

d) BisVCap:Flurbiprofen

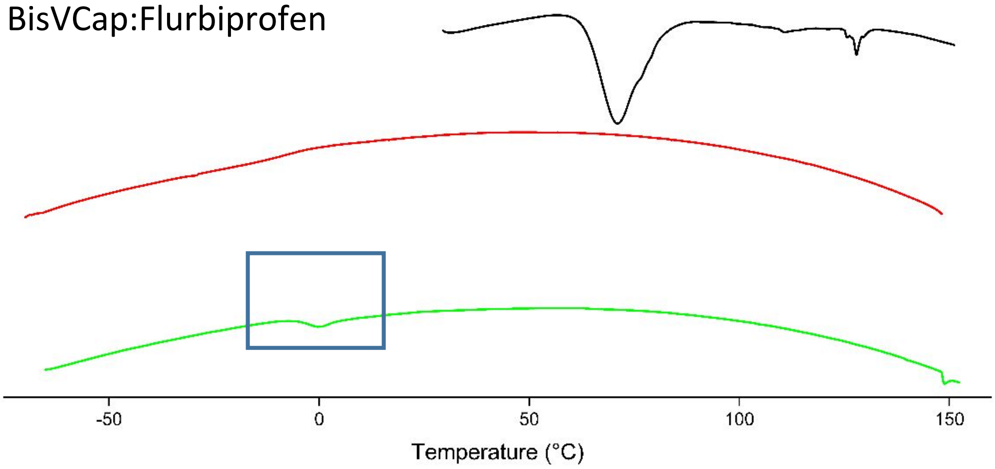

e) BisVCap:Furosemide

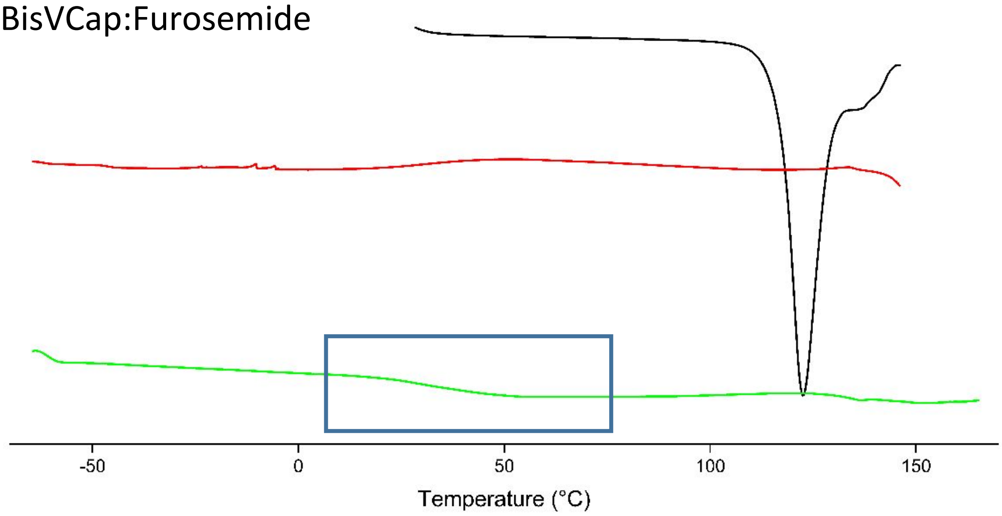

f) BisVCap:Indomethacin

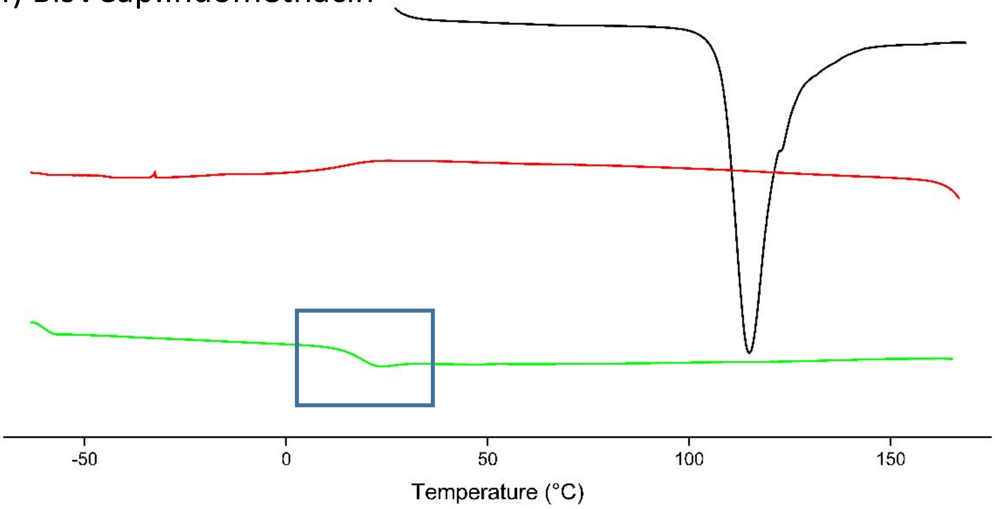

g) BisVCap:Ketoprofen

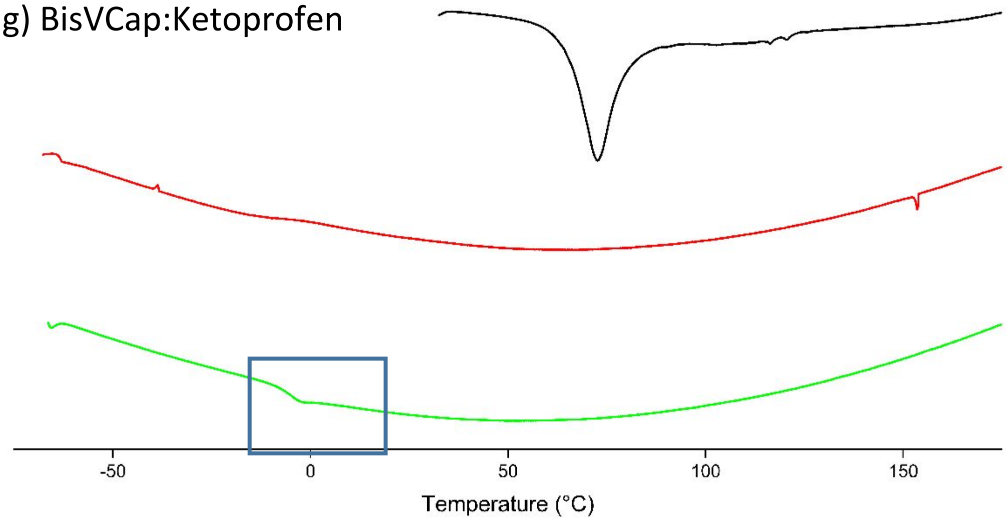

h) BisVCap:Paracetamol

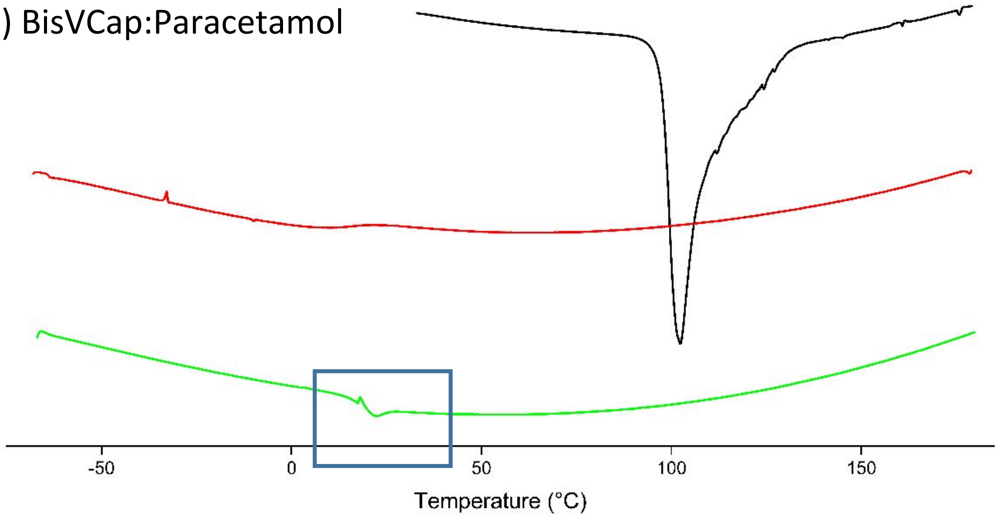

i) BisVCap:Phenobarbital

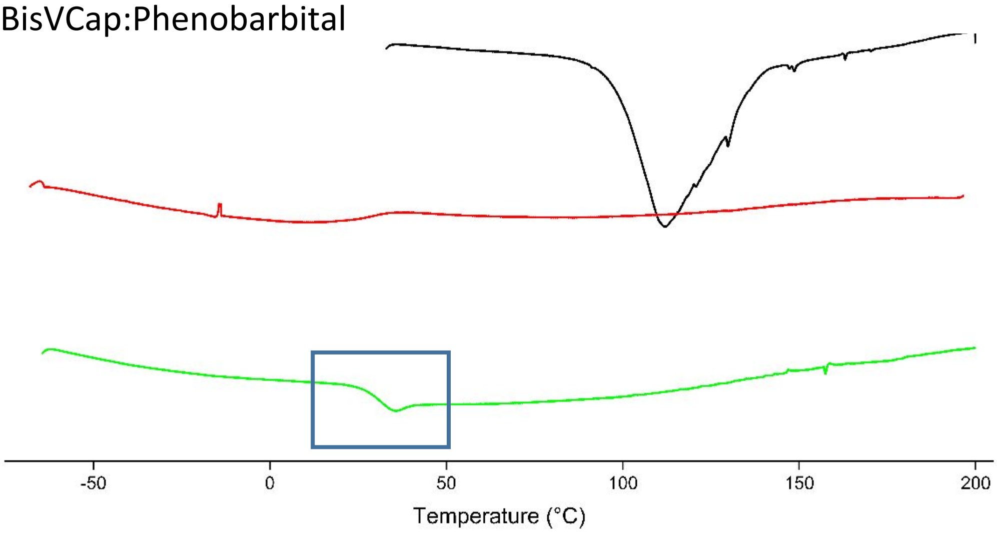

j) BisVCap:Simvastatin

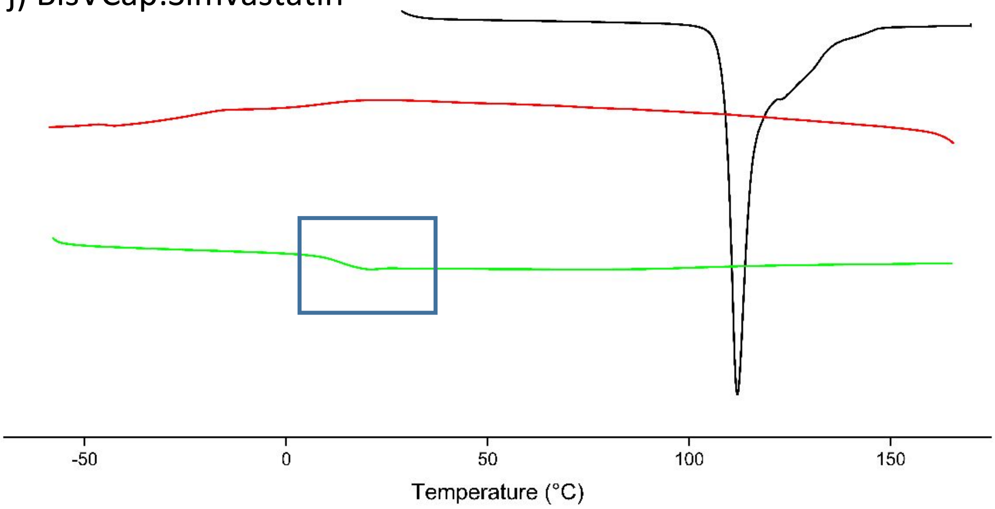

Figure S1: The heat/cool/heat DSC thermograms of 1:1 mixtures of the APIs with bisVCap with exotherms up. The different APIs are a) aspirin, b) chloramphenicol, c) chlorpropamide, d) flurbiprofen, e) furosemide, f) indomethacin, g) ketoprofen, h) paracetamol, i) phenobarbital and j) simvastatin. The black trace displays the initial heat cycle, followed by the cooling cycle in red and the second heat cycle in green. The T_g is highlighted with a blue box on each thermogram.

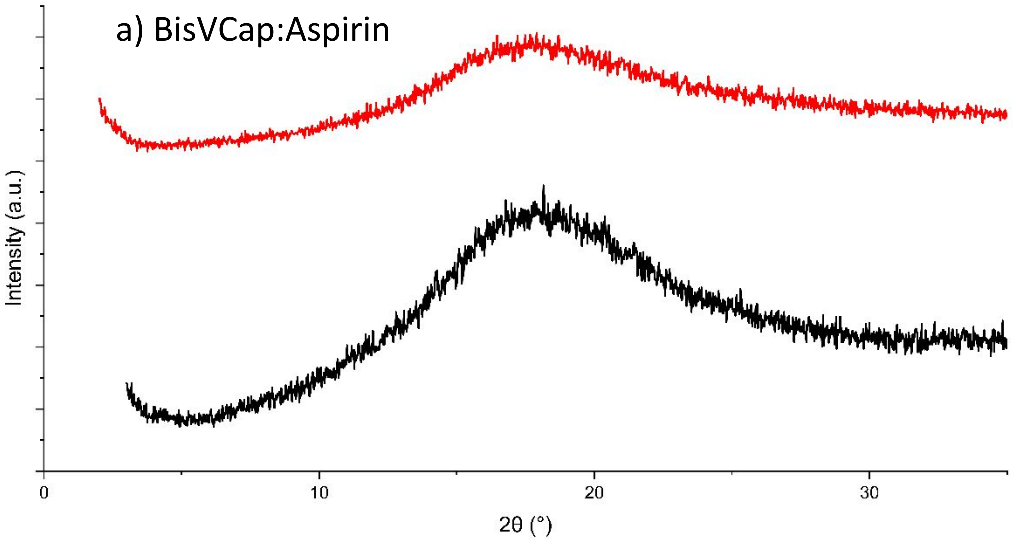
a) BisVCap:Aspirin
Intensity (a.u.)
0
10
20
30
2θ (°)

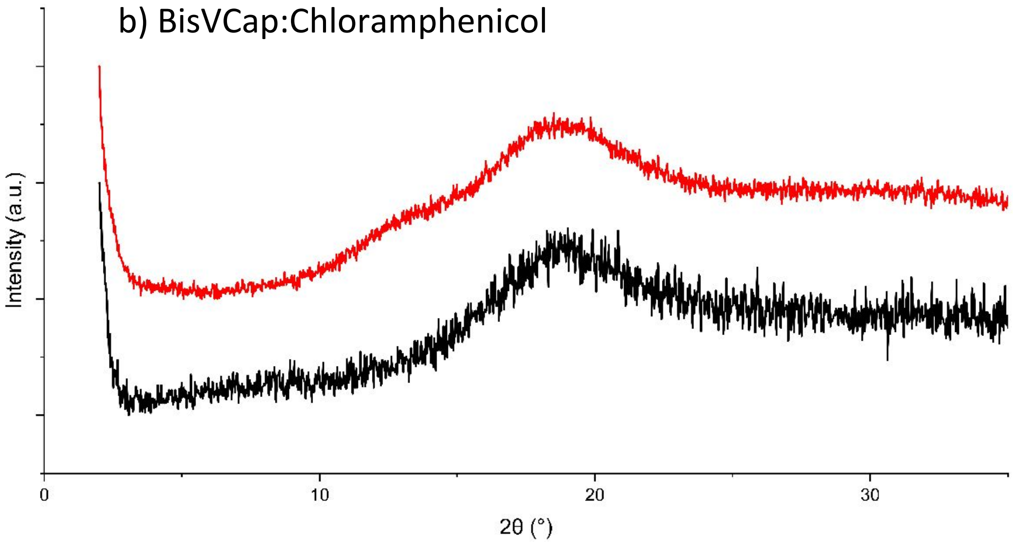
b) BisVCap:Chloramphenicol
Intensity (a.u.)
0
10
20
30
2θ (°)

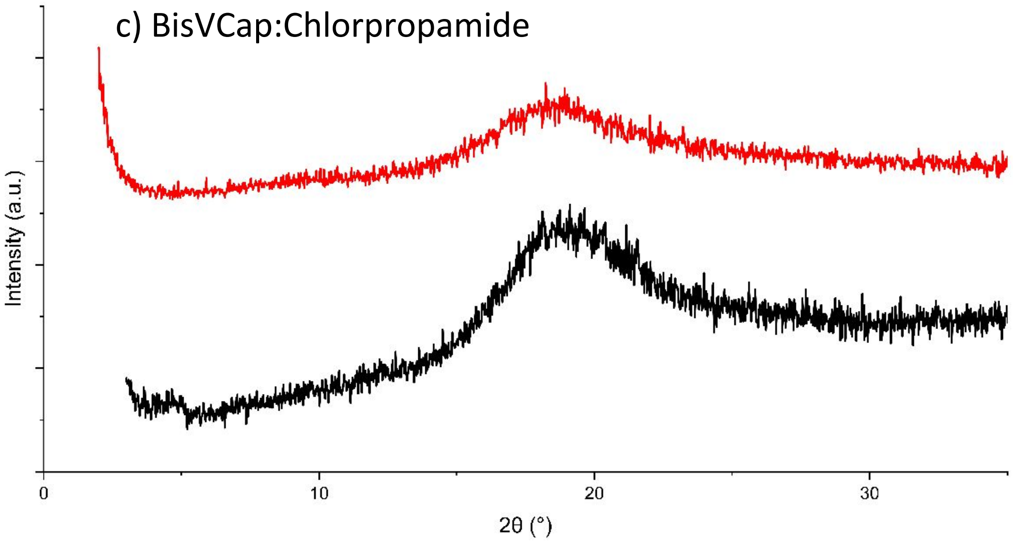
c) BisVCap:Chlorpropamide
Intensity (a.u.)
0
10
20
30
2θ (°)

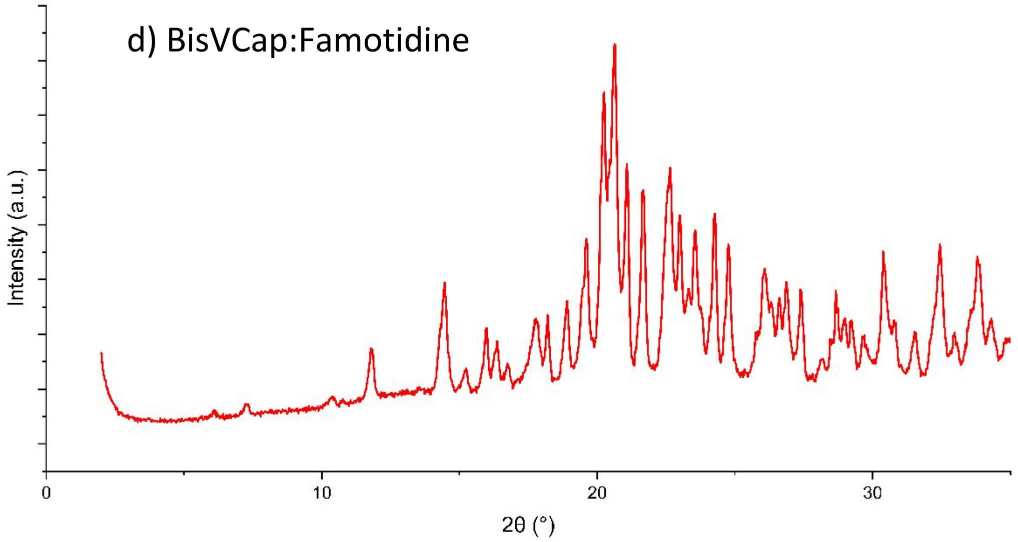
d) BisVCap:Famotidine
Intensity (a.u.)
0
10
20
30
2θ (°)

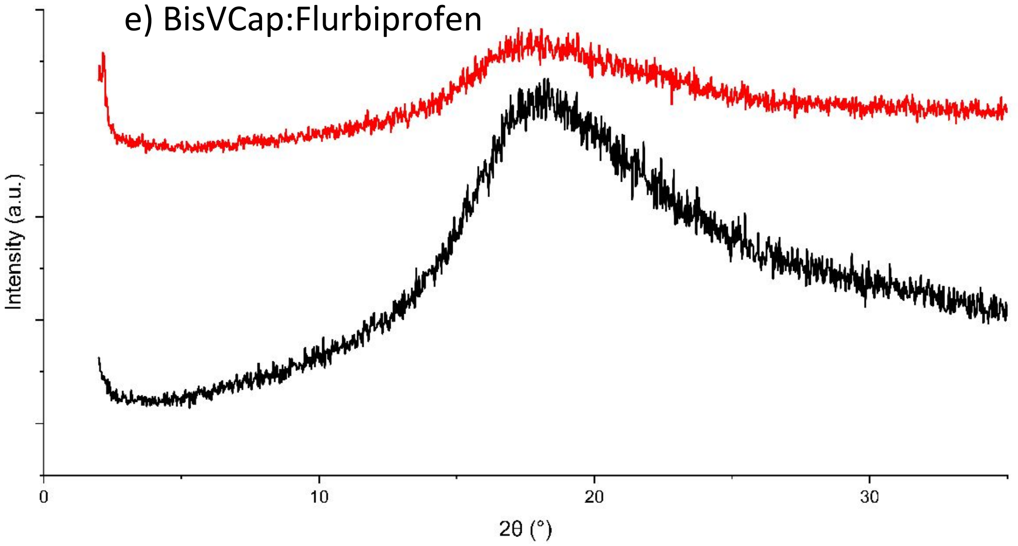
e) BisVCap:Flurbiprofen
Intensity (a.u.)
0
10
20
30
2θ (°)

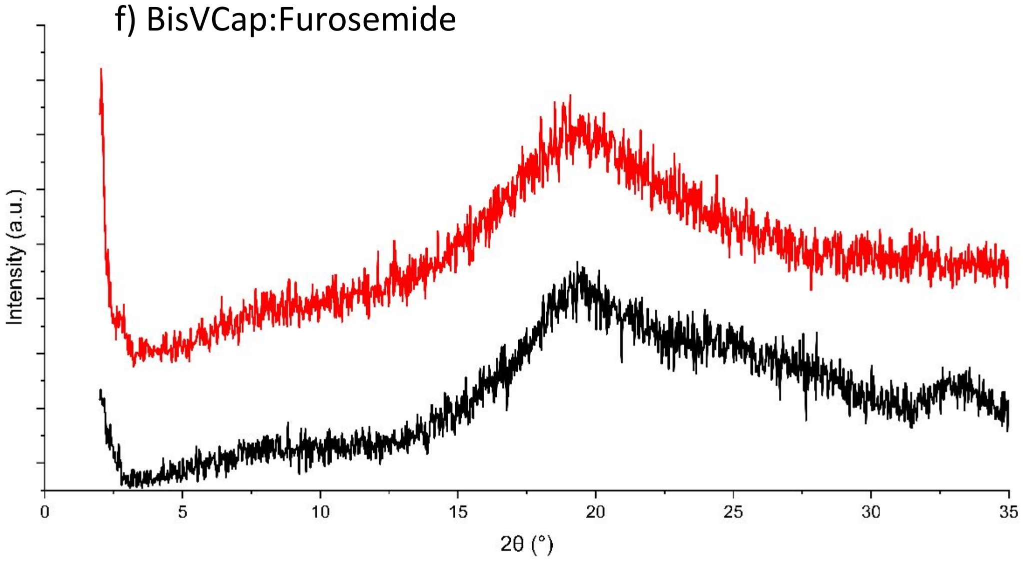
f) BisVCap:Furosemide
Intensity (a.u.)
0
5
10
15
20
25
30
35
2θ (°)

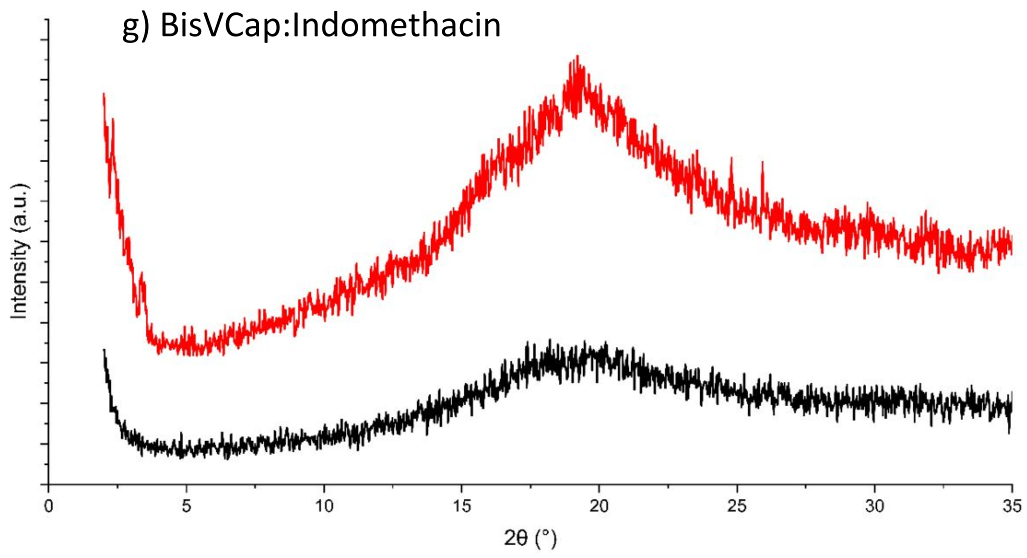
g) BisVCap:Indomethacin
Intensity (a.u.)
0
5
10
15
20
25
30
35
2θ (°)

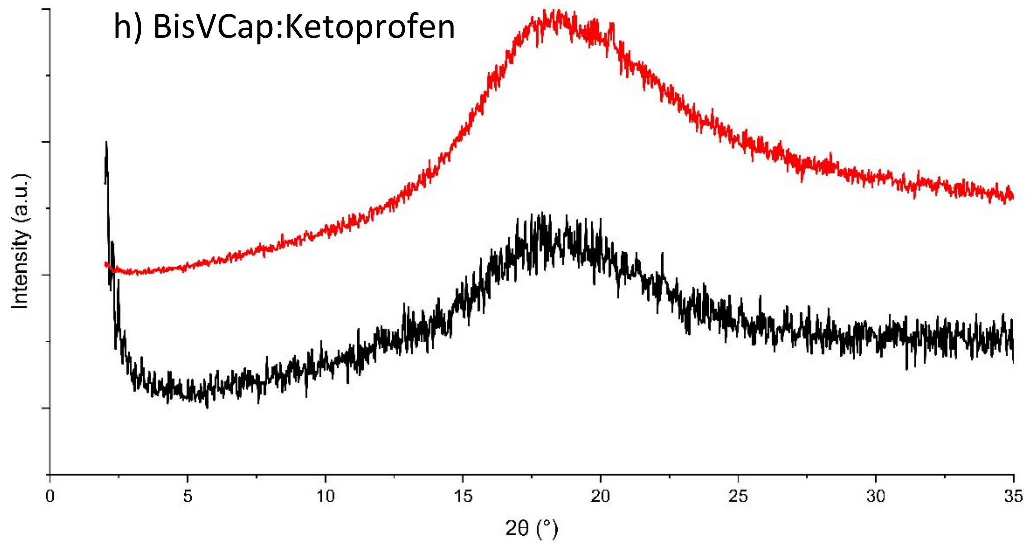
h) BisVCap:Ketoprofen
Intensity (a.u.)
0
5
10
15
20
25
30
35
2θ (°)

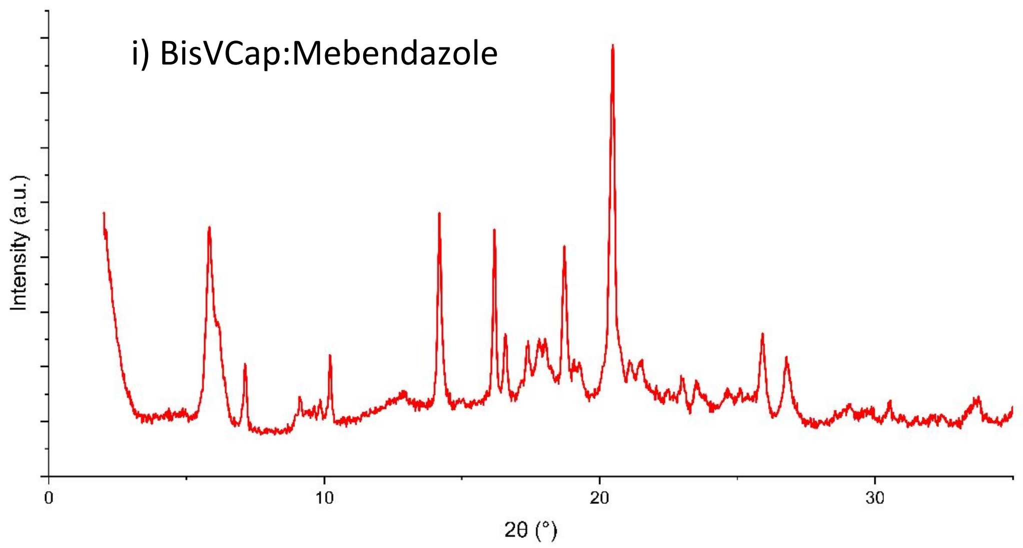
i) BisVCap:Mebendazole
Intensity (a.u.)
0
10
20
30
2θ (°)

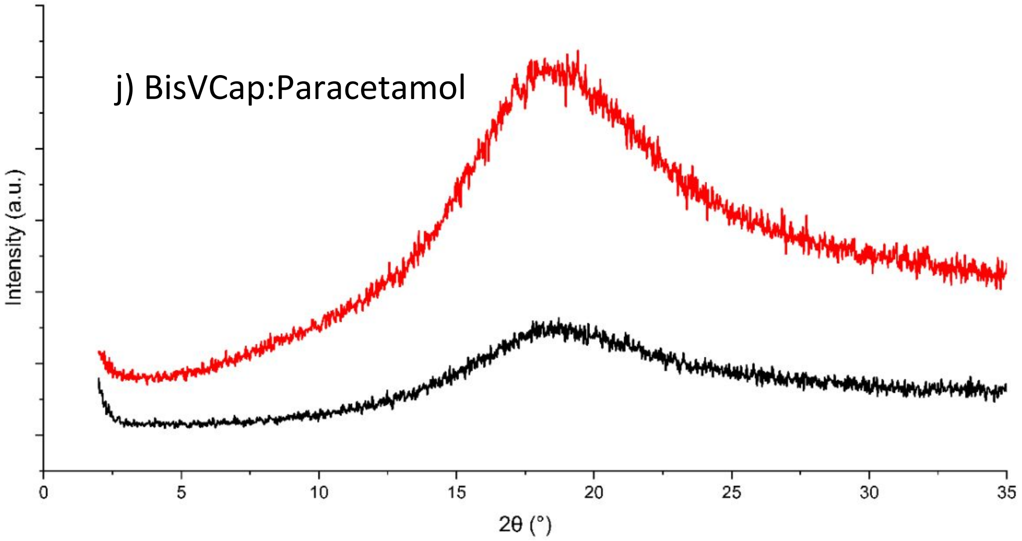
j) BisVCap:Paracetamol
Intensity (a.u.)
0
5
10
15
20
25
30
35
2θ (°)

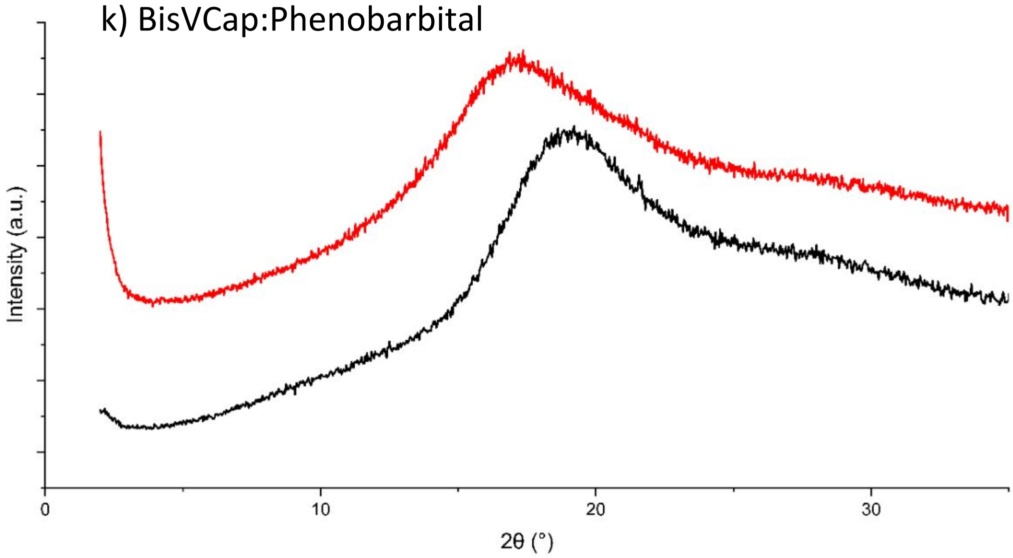
k) BisVCap:Phenobarbital
Intensity (a.u.)
0
10
20
30
2θ (°)

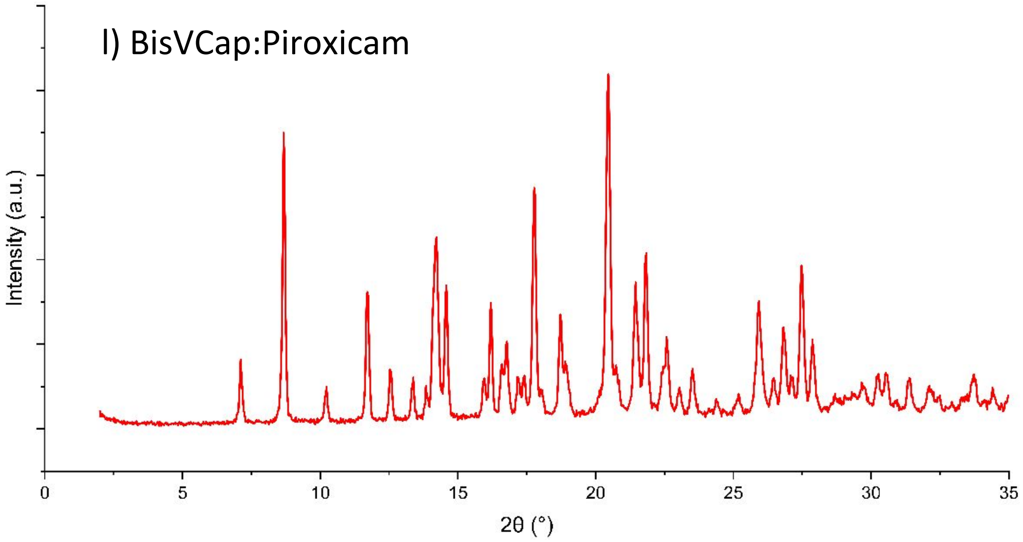
l) BisVCap:Piroxicam
Intensity (a.u.)
0
5
10
15
20
25
30
35
2θ (°)

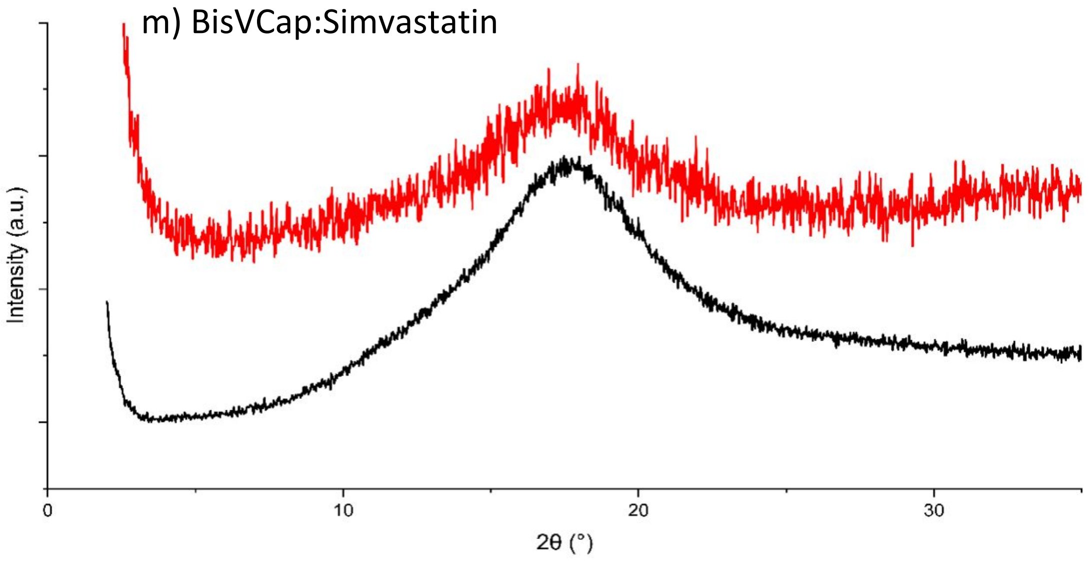

Figure S2: The XRPD diffractograms for the COAM screen of bisVCap with 13 APIs. The CM samples are shown in black and the RSE samples are in red. The different APIs are a) aspirin, b) chloramphenicol, c) chlorpropamide, d) famotidine, e) flurbiprofen, f) furosemide, g) indomethacin, h) ketoprofen, i) mebendazole, j) paracetamol, k) phenobarbital, l) piroxicam, and m) simvastatin. Famotidine, mebendazole and piroxicam only have an RSE trace due to the system decomposing when undergoing CM.

a) BisVCap:Aspirin

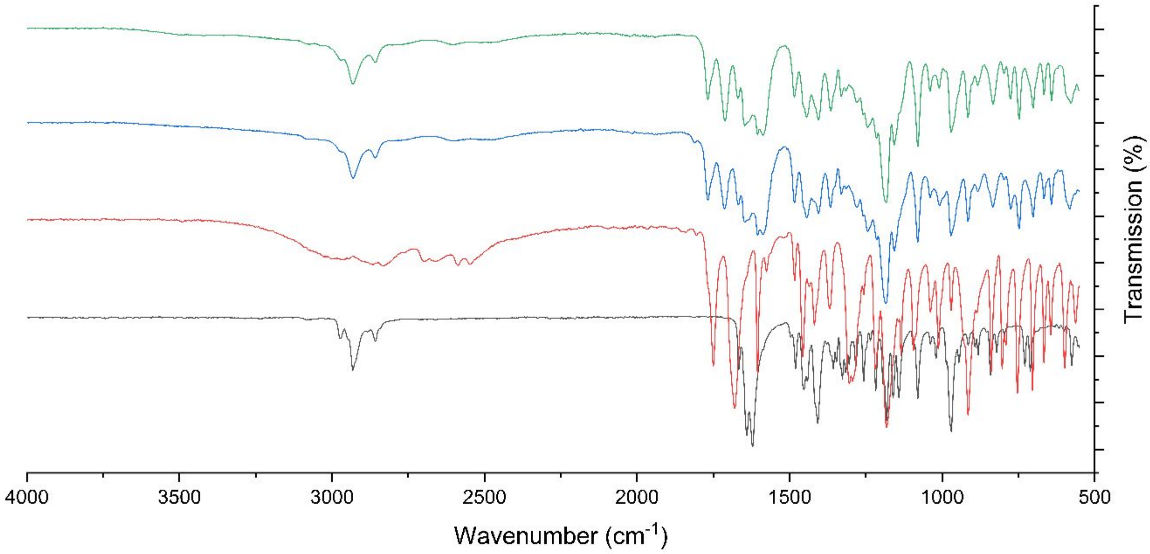

b) BisVCap:Chloramphenicol

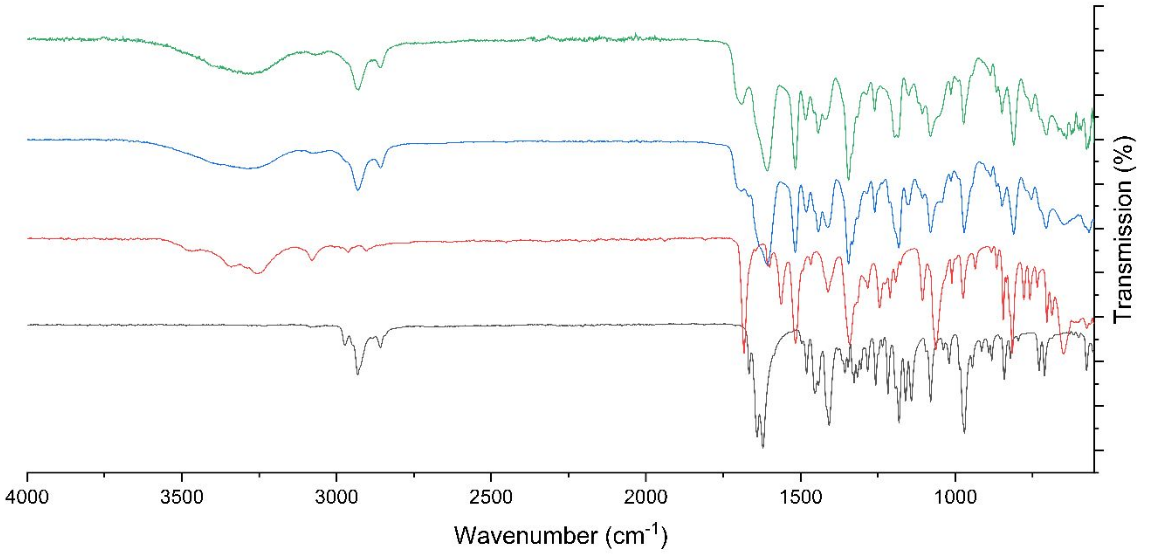

c) BisVCap:Chlorpropamide

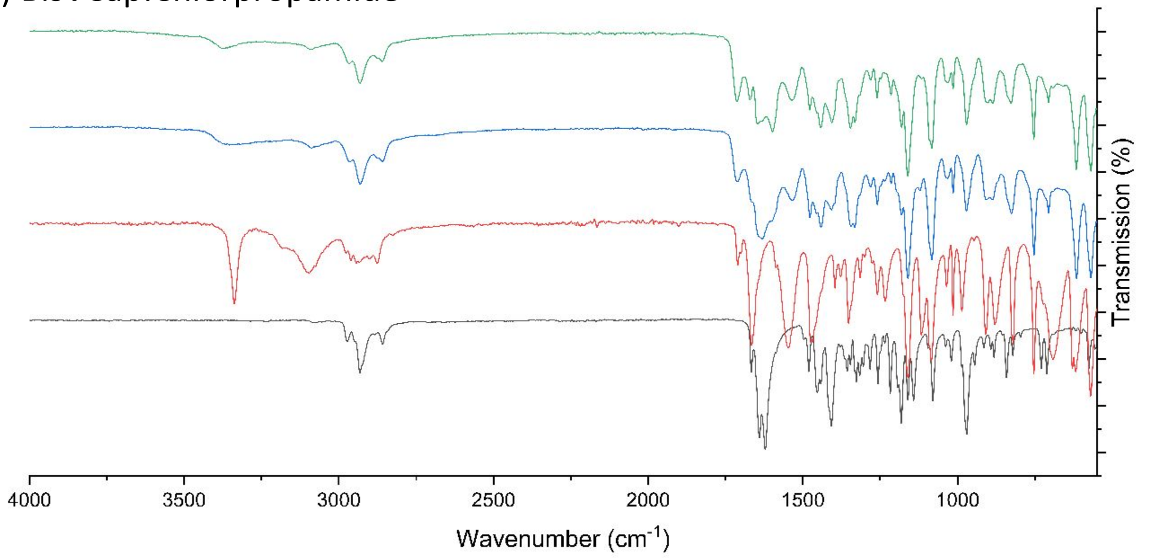

d) BisVCap:Famotidine

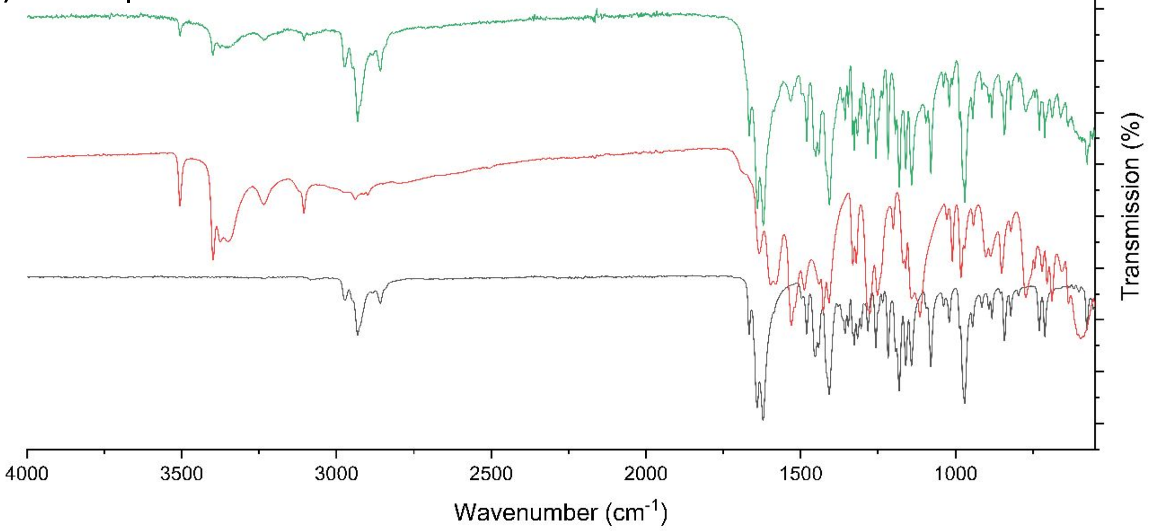

e) BisVCap:Flurbiprofen

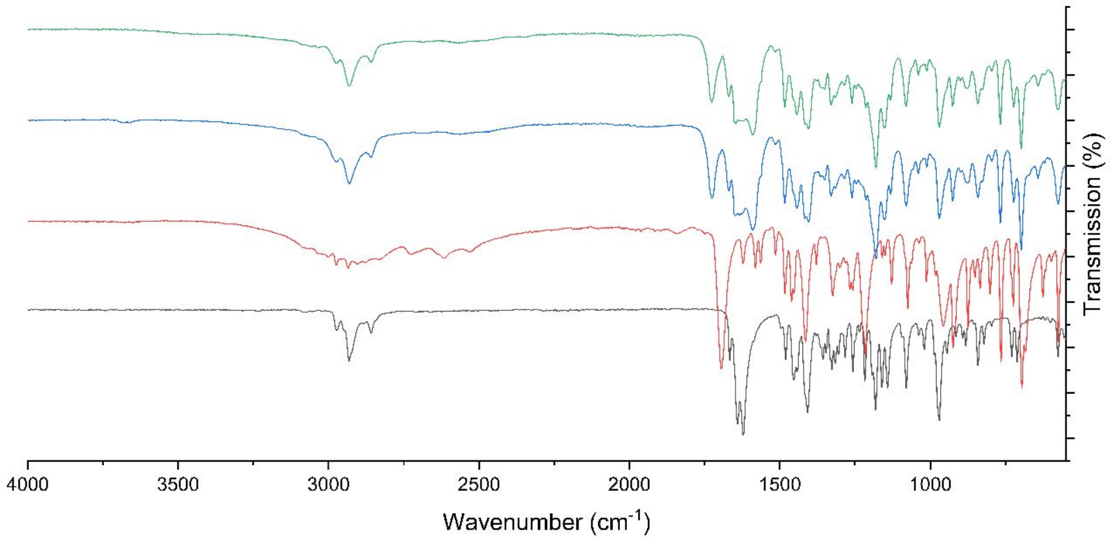

f) BisVCap:Furosemide

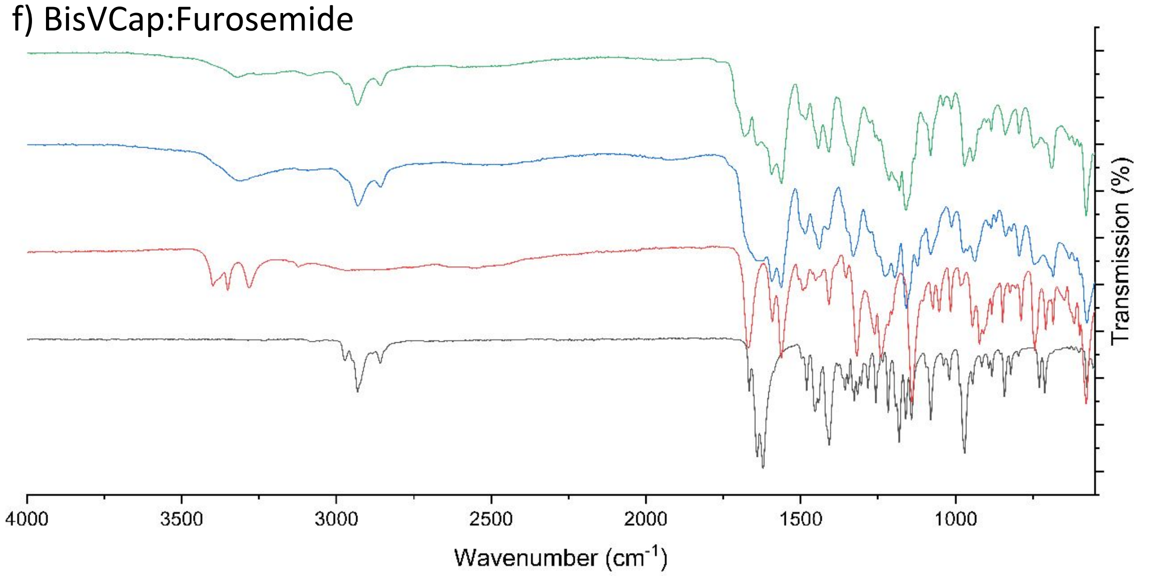

g) BisVCap:Indomethacin

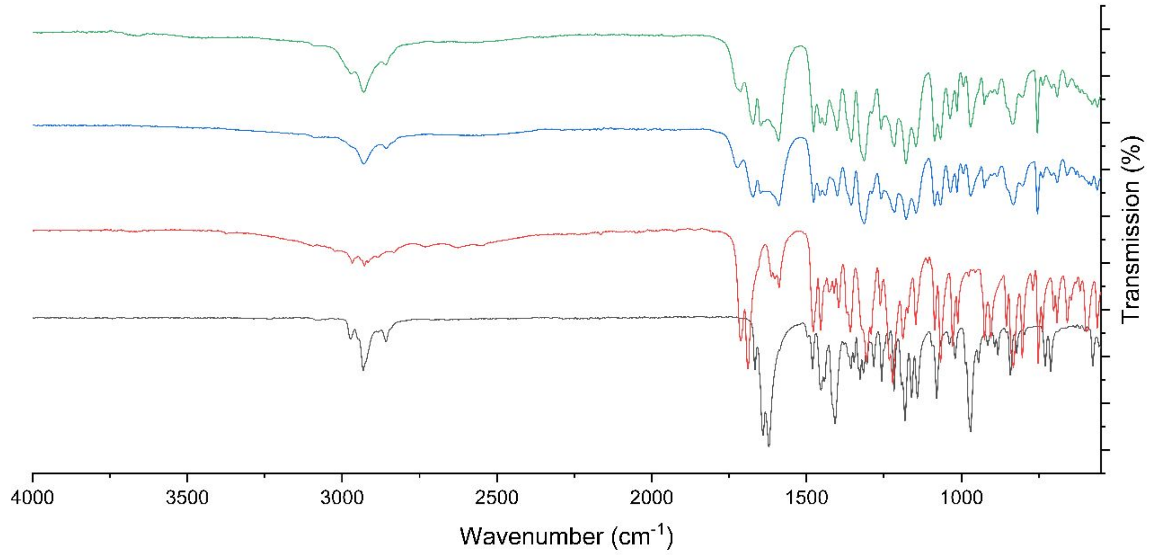

h) BisVCap:Ketoprofen

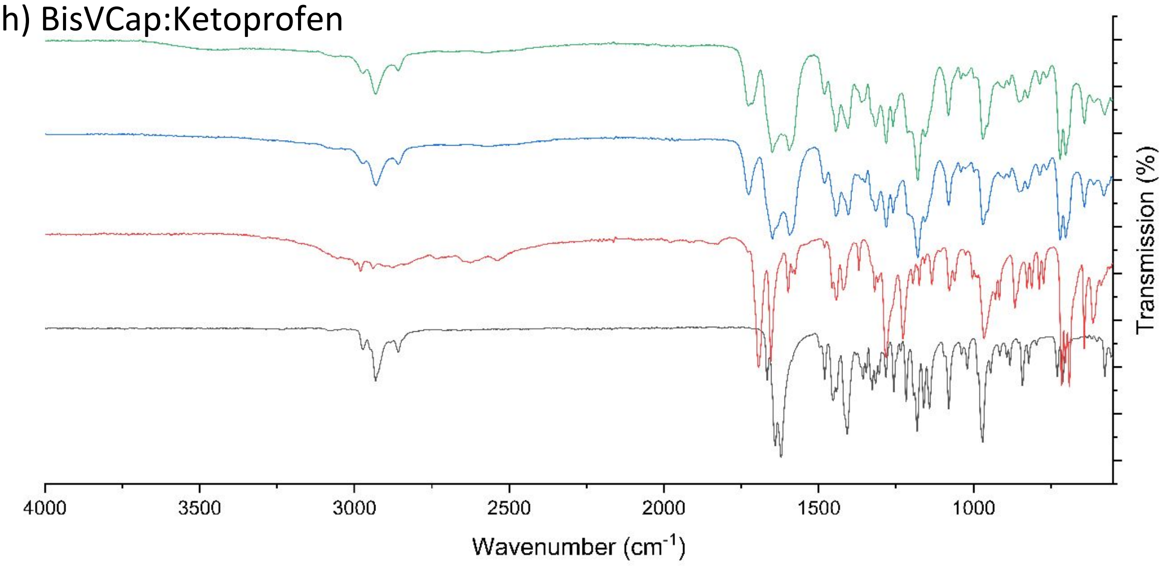

i) BisVCap:Mebendazole

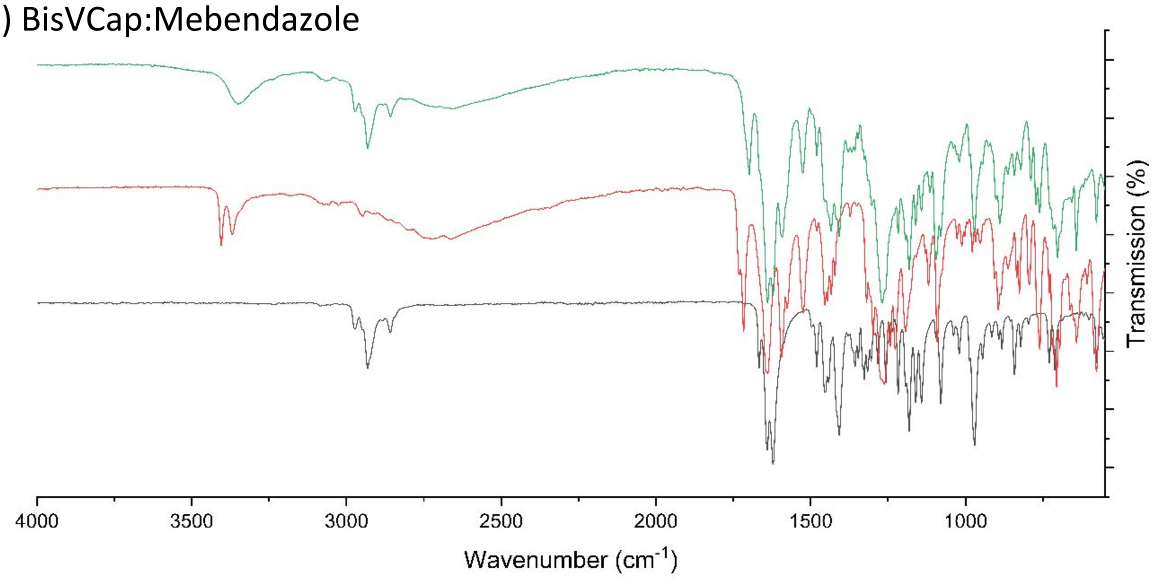

j) BisVCap:Paracetamol

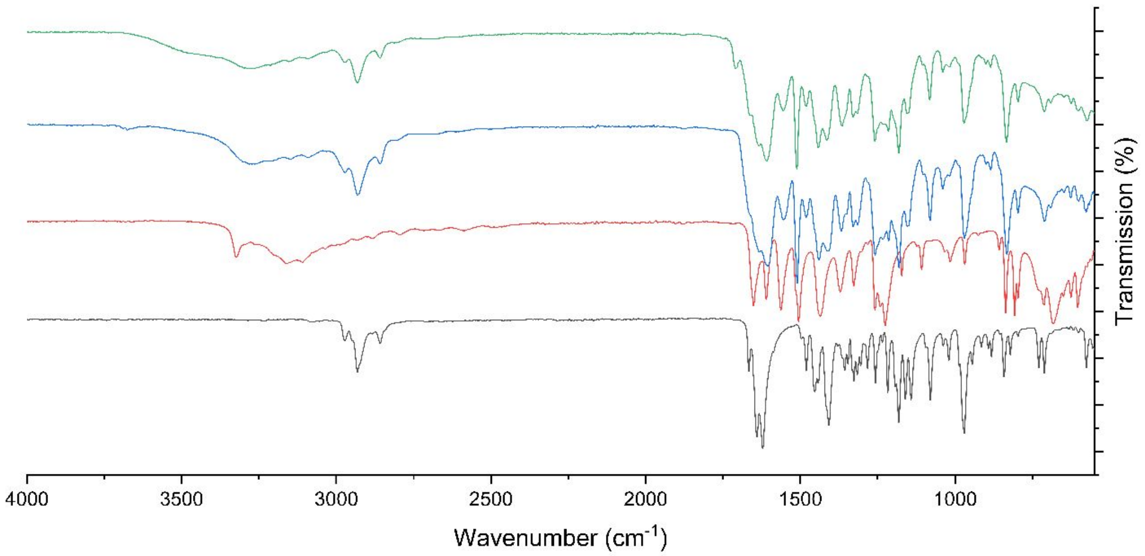

k) BisVCap:Phenobarbital

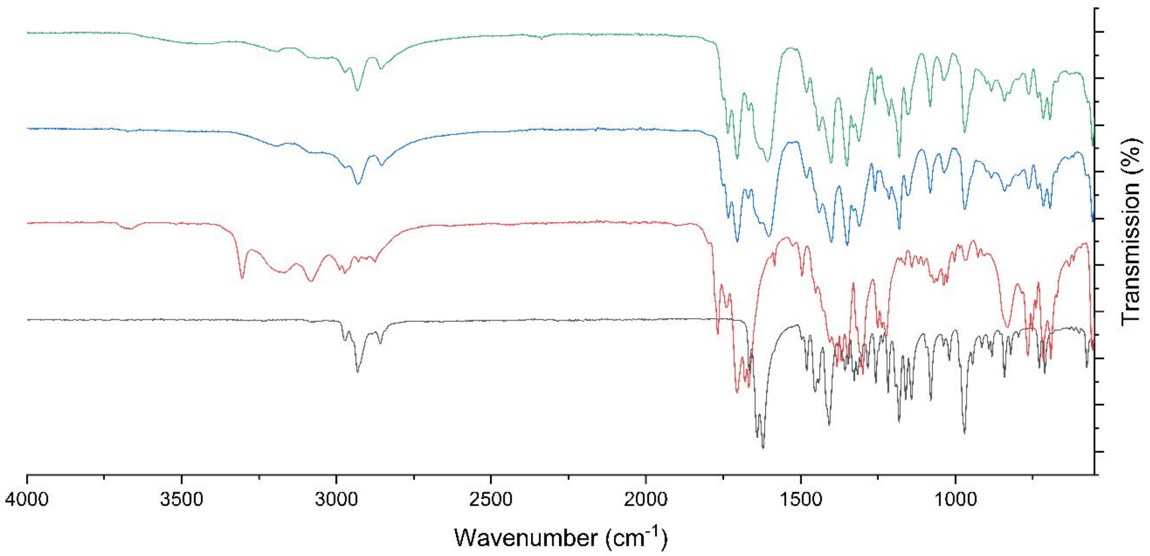

l) BisVCap:Piroxicam

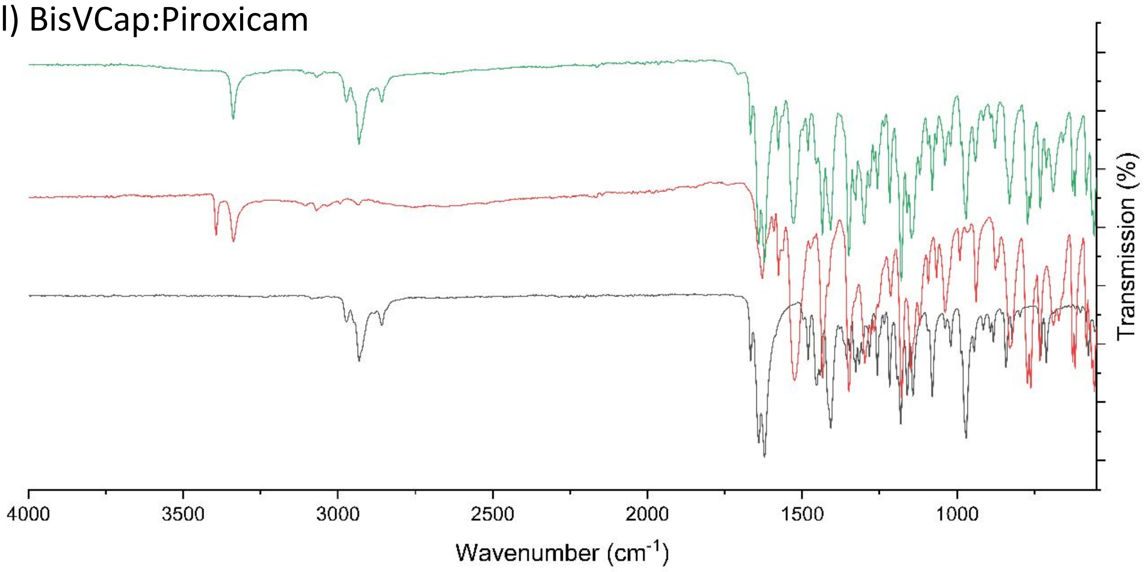

m) BisVCap:Simvastatin

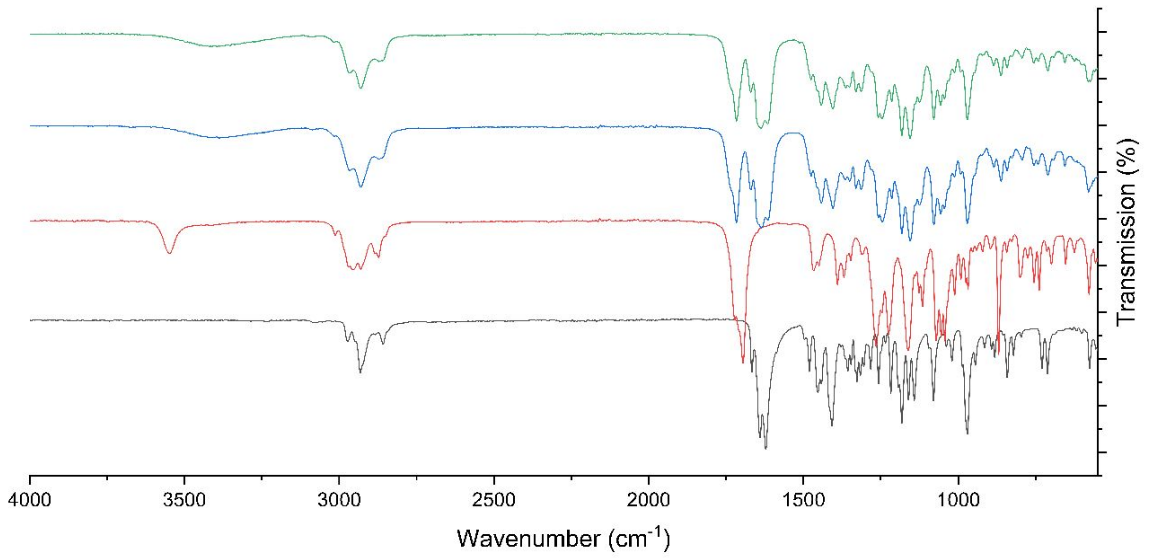

Figure S3: The FTIR spectra for the COAM screen of bisVCap with 13 APIs. BisVCap is shown in black, the pure API in red, the CM sample in blue and the RSE sample in green. The different APIs are a) aspirin, b) chloramphenicol, c) chlorpropamide, d) famotidine, e) flurbiprofen, f) furosemide, g) indomethacin, h) ketoprofen, i) mebendazole, j) paracetamol, k) phenobarbital, l) piroxicam, and m) simvastatin. The famotidine, mebendazole and piroxicam spectra only display an RSE sample due to the system decomposing when undergoing CM.

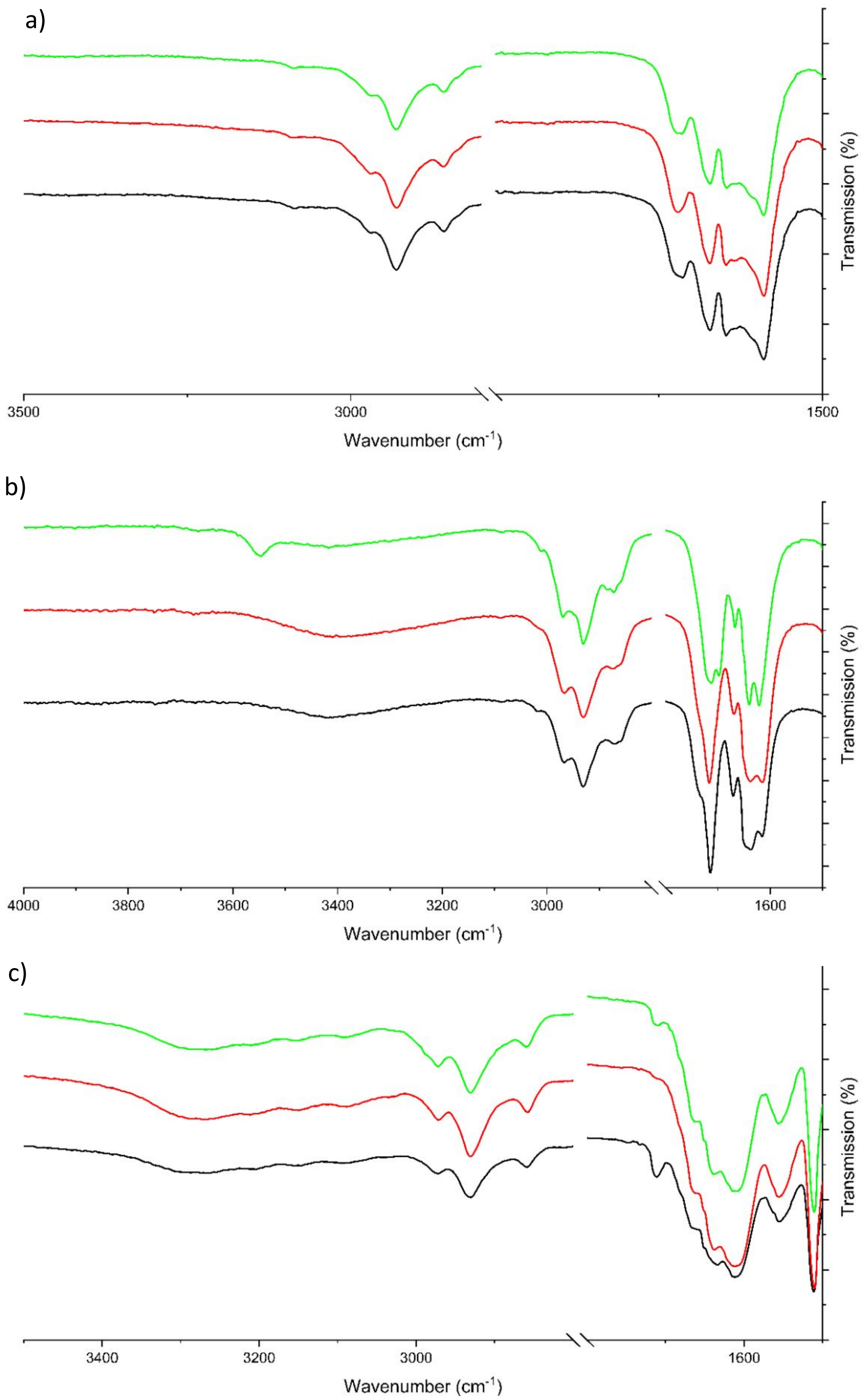

Figure S4: The FTIR spectra of COAM samples of bisVCap with a) indomethacin, b) simvastatin and c) paracetamol. The initial COAM sample made by RSE is shown in black. The FTIR spectra are shown after two weeks when stored at ~20 °C (red) and 3 °C (green).

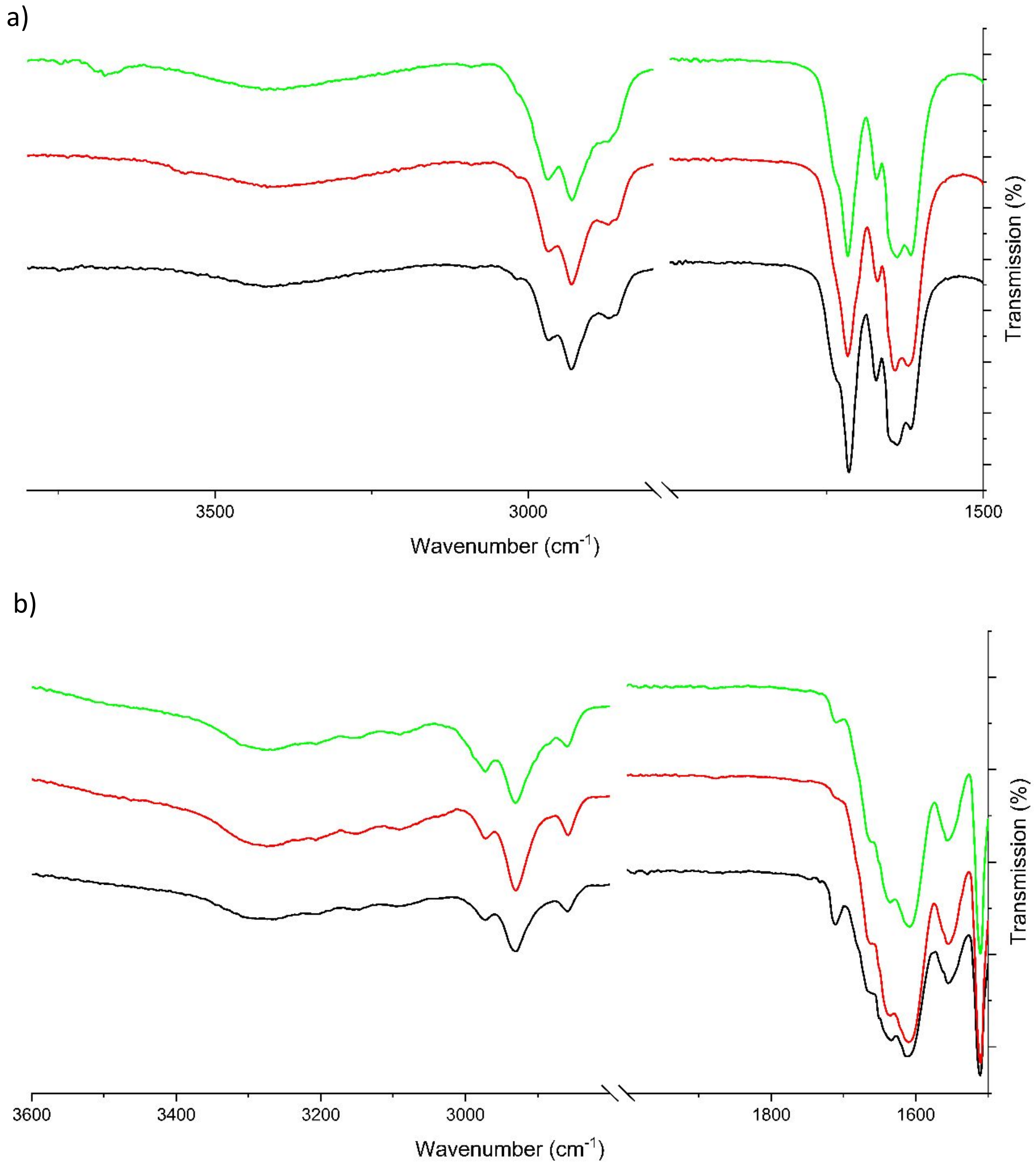

Figure S5: The FTIR spectra of COAM samples of bisVCap with a) simvastatin and b) paracetamol. The initial FTIR spectra after RSE is shown in black. The FTIR spectra are shown after one week when stored at ~20 °C (red) and 3 °C (green).

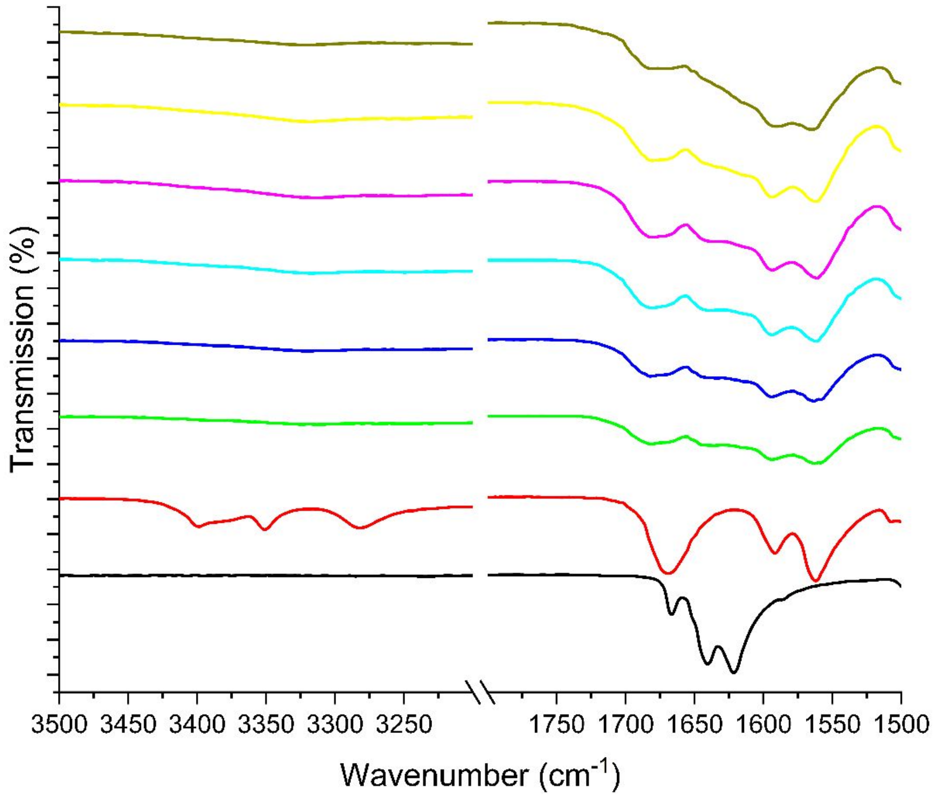

Figure S6: The FTIR spectra of a COAM bisVCap furosemide system made by RSE. Pure bisVCap is shown in black, pure furosemide in red and the initial bisVCap furosemide COAM system in green. The system was stored for seven days at 0% RH (blue), 11% RH (cyan), 33% RH (pink), 75% RH (yellow) and 100% RH (brown).

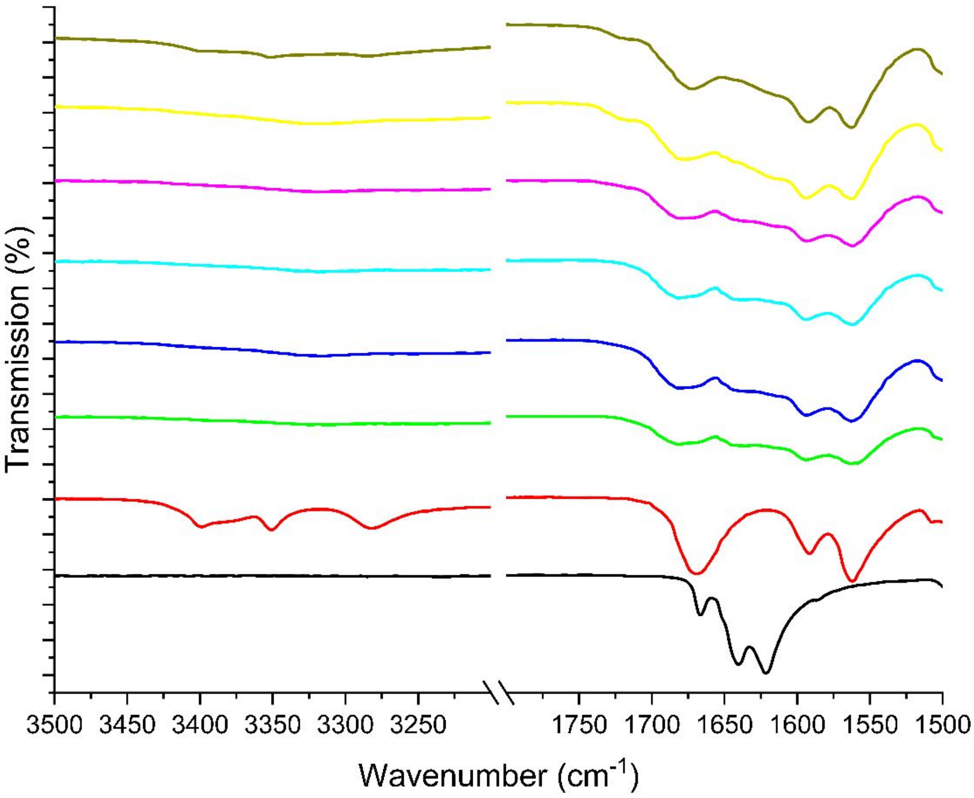

Figure S7: The FTIR spectra of a COAM bisVCap furosemide system made via RSE. Pure bisVCap is shown in black, pure furosemide in red and the initial bisVCap furosemide COAM system in green. The system was stored for 28 days at 0% RH (blue), 11% RH (cyan), 33% RH (pink), 75% RH (yellow) and 100% RH (brown).
